# Feasibility of eliminating visceral leishmaniasis from the Indian subcontinent: explorations with a set of deterministic age-structured transmission models

**DOI:** 10.1186/s13071-016-1292-0

**Published:** 2016-01-19

**Authors:** Epke A. Le Rutte, Luc E. Coffeng, Daniel M. Bontje, Epco C. Hasker, José A. Ruiz Postigo, Daniel Argaw, Marleen C. Boelaert, Sake J. De Vlas

**Affiliations:** Department of Public Health, Erasmus MC, University Medical Center Rotterdam, PO Box 2040, 3000 CA Rotterdam, The Netherlands; Institute of Tropical Medicine, Nationalestraat 155, 2000 Antwerp, Belgium; World Health Organization, Avenue Appia 20, 1211 Geneva, Switzerland

**Keywords:** Visceral leishmaniasis, Elimination, Mathematical modelling, Indoor residual spraying, Indian subcontinent, Neglected tropical disease, Kala-azar, Reservoir of infection, Sandfly, Transmission dynamics

## Abstract

**Background:**

Visceral leishmaniasis (VL) is a neglected tropical disease transmitted by sandflies. On the Indian subcontinent (ISC), VL is targeted for elimination as a public health problem by 2017. In the context of VL, the elimination target is defined as an annual VL incidence of <1 per 10,000 capita at (sub-)district level. Interventions focus on vector control, surveillance and on diagnosing and treating VL cases. Many endemic areas have not yet achieved optimal control due to logistical, biological as well as technical challenges. We used mathematical modelling to quantify VL transmission dynamics and predict the feasibility of achieving the VL elimination target with current control strategies under varying assumptions about the reservoir of infection in humans.

**Methods:**

We developed three deterministic age-structured transmission models with different main reservoirs of infection in humans: asymptomatic infections (model 1), reactivation of infection after initial infection (model 2), and post kala-azar dermal leishmaniasis (PKDL; model 3). For each model, we defined four sub-variants based on different assumptions about the duration of immunity and age-patterns in exposure to sandflies. All 12 model sub-variants were fitted to data from the KalaNet study in Bihar (India) and Nepal, and the best sub-variant was selected per model. Predictions were made for optimal and sub-optimal indoor residual spraying (IRS) effectiveness for three different levels of VL endemicity.

**Results:**

Structurally different models explained the KalaNet data equally well. However, the predicted impact of IRS varied substantially between models, such that a conclusion about reaching the VL elimination targets for the ISC heavily depends on assumptions about the main reservoir of infection in humans: asymptomatic cases, recovered (immune) individuals that reactivate, or PKDL cases.

**Conclusions:**

Available data on the impact of IRS so far suggest one model is probably closest to reality (model 1). According to this model, elimination of VL (incidence of <1 per 10,000) by 2017 is only feasible in low and medium endemic settings with optimal IRS. In highly endemic settings and settings with sub-optimal IRS, additional interventions will be required.

**Electronic supplementary material:**

The online version of this article (doi:10.1186/s13071-016-1292-0) contains supplementary material, which is available to authorized users.

## Background

On the Indian subcontinent (ISC), visceral leishmaniasis (VL) is caused by the protozoan *Leishmania donovani*, which is transmitted by the peri-domestic female sandfly, *Phlebotomus argentipes*. VL is a neglected tropical disease (NTD) [1] with about 300 million people at risk globally, mainly affecting the poorest of the poor in rural areas. Two thirds of the estimated global 200,000 to 400,000 new VL cases per year occur on the ISC [2]. Furthermore, over 20,000 deaths per year on the ISC are attributed to VL, making it the deadliest parasitic infection in the world after malaria [3, 4]. Humans are considered the only host for *L. donovani* on the ISC, whereas in the rest of the world VL is both anthroponotic and zoonotic, and can also be caused by *L. infantum* [3]. Only a small fraction of the people that become infected develop clinical symptoms, while most remain asymptomatic, nonetheless carrying the parasite [5]. People that develop symptoms of VL, also known as kala-azar (KA), display signs of fever, weight loss, anaemia and splenomegaly, and eventually die if left untreated [6, 7]. It is estimated that about one to five percent of successfully treated VL cases on the ISC develop post-kala-azar dermal leishmaniasis (PKDL), a self-healing skin disease which may last for several years [8–10]. *L. donovani* infection can be diagnosed by – among other methods –testing of peripheral blood for parasite DNA by means of polymerase chain reaction (PCR), and by testing for antibodies using the direct agglutination test (DAT, a marker for humoral immune response indicating current or recent infection).

Even though attention for VL has grown over the past decade, its transmission dynamics are still not completely understood. For instance, little is known about the role and duration of acquired immunity after infection, the infectiveness of different disease stages towards the sandfly, and natural sandfly behavior. The observation of low and infrequent numbers of symptomatic VL cases, which by themselves are not sufficient to sustain transmission, suggests the presence of a parasite reservoir, which is also supported by high proportions of PCR+ individuals [11]. Even though the parasite has been found in domestic animals, their role in transmission on the ISC has not been established [12], and therefore humans remain the only confirmed reservoir of the parasite on the ISC. Potential human reservoirs of infection (apart from the low number of symptomatic cases) are asymptomatic infections, persons in whom a past infection reactivates, PKDL cases, or a mixture of these.

In 2012, WHO developed the first NTD 2020 Roadmap that contains targets for the elimination and control of VL [13]. That same year, the London Declaration was signed by several partners from the public and private sector, to support the 2020 WHO Roadmap targets through advocacy, pharmaceutical supplies and research funding [14]. On the ISC, the target is to eliminate VL as a public health problem by or before the end of 2017, where elimination is defined as an annual incidence of VL of <1 per 10,000 capita at sub-district-levels in Bangladesh and India; and at district-level in Bhutan and Nepal [15]. In the rest of the world, the WHO target is 100 % detection and treatment of all VL cases. In the ideal situation of meeting the WHO targets for VL, the global impact (relative to the counterfactual had the pre-control situation in the year 1990 continued unabated) has been estimated at 2.4 million averted deaths, 140 million averted DALYs, and about 20 billion US dollars saved between 2011 and 2030 [16, 17].

The governments of the ISC-countries have committed themselves to achieving the elimination target by implementing different interventions. These are mainly focused on two approaches: (1) early diagnosis of symptomatic cases followed by effective case management, which prevents disability and death, and reduces the presence of infective individuals; and (2) vector control to reduce or interrupt transmission [3]. Indoor residual spraying (IRS) of human dwellings and cattle sheds with long lasting insecticides such as DDT is currently the most important and widely implemented form of vector control. To a lesser extent, insecticide–treated bed nets, environmental management and personal protection are also being implemented [18, 19]. Although indoor spraying campaigns on the ISC have been scaled up over the last years, not all regions have yet achieved effective IRS programs due to various challenges such as limited training of spraying teams, poor community acceptance, sandfly resistance to DDT, and the peri-domestic lifestyle of the sandfly [19–24].

Here, we focus on the following research question: is it technically feasible to achieve the WHO VL elimination targets on the ISC by 2017 with current IRS strategies and ongoing detection and treatment of cases? To this end, we upgraded the most relevant existing deterministic VL transmission model [25, 26], and developed three age-structured deterministic models representing three potential main parasite reservoirs in humans: (1) asymptomatic cases, (2) recovered (immune) individuals in whom infection reactivates, and (3) cases of PKDL. For each model, we defined four sub-variants with different transmission dynamics: fixed or age-dependent sandfly exposure and a duration of the late recovered ‘immune’ stage of two or five years. All twelve models were quantified using data from the KalaNet study in Bihar (India) and Nepal [27, 28]. With the best sub-variant of each of the three models, we simulated the impact of IRS (optimally and sub-optimally implemented) on VL incidence for three endemic settings to predict the feasibility of achieving the elimination target of <1 VL case per 10,000 capita per year on the ISC.

## Methods

### Model structure

**Fig. 1:**
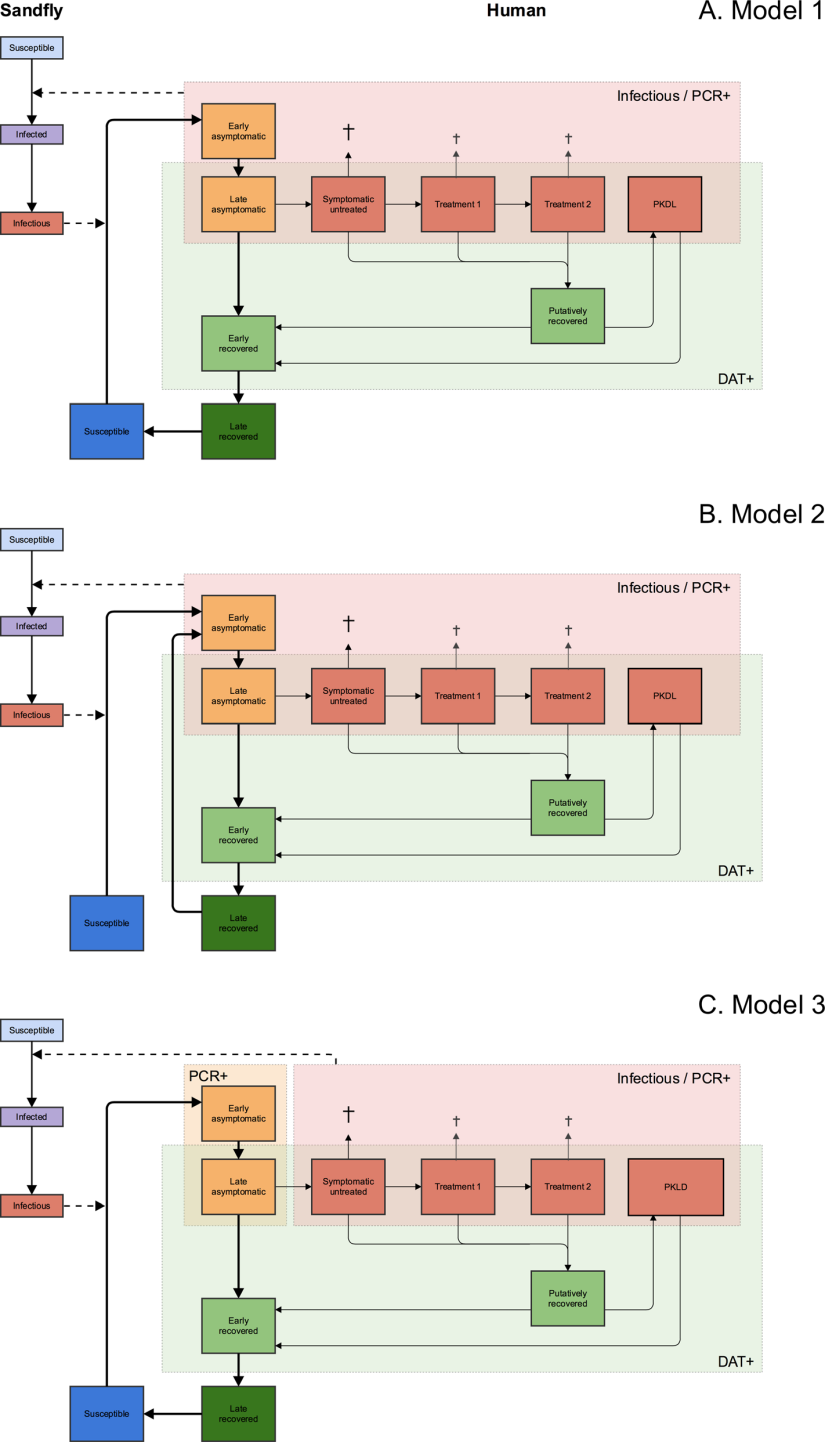
Schematic representation of three model structures. In model 1 (**a**), recovered individuals eventually lose their immunity and become susceptible again to infection through exposure to infective sandflies. In model 2 (**b**), recovered individuals may experience reactivation of their past infection such that they directly re-enter the stage of early asymptomatic infection without requiring exposure to infective sandflies. In model 3, which is identical in structure to model 1 (**c**), only cases of symptomatic infection and PKDL contribute to transmission of infection, and duration of PKDL is three times as long as in model 1

We developed a set of three VL transmission models, each with four sub-variants, based on the general structure of a previous model developed by Stauch and colleagues at Tuebingen University [25, 26]. In all models (see Fig. 1 for schematic representation), we assume that humans are born *susceptible* and, when bitten by an infective sandfly, will move to the stage of *early asymptomatic infection*. We assume that individuals in this stage test positive for parasite DNA using PCR (PCR+), and test negative for antibodies using the direct agglutination test (DAT-). After some time, an infected person will develop antibodies and advance to the stage of *late asymptomatic infection* (PCR+/DAT+). A small fraction of cases with late asymptomatic infection will develop symptoms of VL and enter the stage of *symptomatic untreated* (PCR+/DAT+). While most symptomatic cases will require one or two treatment regimens (stages of *first-line* and *second-line treatment* (PCR+/DAT+), initiated after a detection delay) to clear infection to the extent that parasite DNA is no longer detectable (*putatively recovered* stage, PCR-/DAT+), a small fraction of untreated symptomatic cases will spontaneously clear infection and directly advance to the putatively recovered stage (i.e. non-fatal symptomatic cases that do not turn up in surveillance data because of low severity of disease) [29]. All symptomatic cases are assumed to be at excess risk of dying from VL, with the excess risk being highest in untreated cases. From the putatively recovered stage, a small fraction of individuals may develop *PKDL* (PCR+/DAT+) from which they will eventually recover (spontaneously or with treatment; the exact mechanism of recovery is not specified in the model). However, the majority of cases in the putatively recovered stage advance to the *early recovered* stage (PCR-/DAT+), along with recovered cases of PKDL, and the majority of late asymptomatic infections that do not develop any symptoms and spontaneously clear infection to the extent that parasite DNA is no longer detectable. Eventually, individuals in the early recovered stage will lose their DAT positivity, and enter the *late recovered* stage (PCR-/DAT-), during which they are still immune to new infections. From there, individuals either lose their immunity and become susceptible again to infection through exposure to infective sandflies (model 1), or their past infection reactivates such that they re-enter the stage of early asymptomatic infection without requiring exposure to an infective sandfly (model 2). Model 2 presents a hypothetical but biologically plausible scenario, for example when individuals experience decreased immune-competence during malnutrition or co-infection (e.g. HIV) [30]. In terms of structure, model 1 is the most similar to the model by Stauch et al. [25].

In each model, infection is transmitted between humans by bites of female sandflies (we do not consider male sandflies, which only feed on plant sugars). We define the sandfly population in terms of sandflies per human, a quantity that incorporates sandfly density, the unknown ratio of blood meals taken on human and animals, and the unknown (average) vector competence of sandflies. The sandfly population is partitioned into 3 compartments; all sandflies are born *susceptible* and after feeding on an infective human, they become *infected* with some probability depending on the infectiveness of the human stage of infection. After an incubation period, infected sandflies become *infective* and may infect susceptible humans. We assume no excess mortality among infected sandflies. IRS is assumed to reduce the sandfly density and consequently, human exposure to sandfly bites.

In models 1 and 2, all PCR+ human stages (asymptomatic and symptomatic infection, and PKDL) are considered to be infective towards sandflies, with early asymptomatic cases being half as infective as late asymptomatic cases (as assumed by Stauch et al. [25]). Infectiveness of untreated clinical cases is set at 1.0, treated patients and PKDL have an infectiveness of 0.5, and that of asymptomatic cases is estimated. In model 3, which is identical in structure to model 1, only cases of symptomatic infection and PKDL are assumed to contribute to transmission [31], with PKDL having a higher (estimated) infectiveness than in models 1 and 2. Further, in model 3 we set the duration of PKDL to thrice as long as in model 1, based on expert opinion, assuming that there is a larger spectrum of PKDL severities than currently recognized, of which undiagnosed forms also contribute to transmission. Model 3 can be considered an extreme variant of model 1. A model variant in which only symptomatic human cases (VL and regular PKDL) are infective towards the sandfly, could not be fitted to data on the infection prevalence in sandflies under the assumptions of an endemic equilibrium and homogeneous mixing of human and sandfly populations (Additional file 1, section 5). This indicates that, in order to meet the infection prevalence in sandflies (Table A1-2 in Additional file 1, section 3), there has to be an additional reservoir of infection in humans that are PCR+, which could be in asymptomatic individuals (models 1 and 2), or in long lasting PKDL cases (model 3).

The transmission model was defined in terms of a system of ordinary differential equations (ODE; see Additional file 1, section 2). Hence, we assumed that all transitions between stages take place at constant rates, leading to exponentially distributed durations of stages. However, because the human demography on the ISC cannot be well approximated by the assumption of a stable human population size and exponential human survival (as applied by Stauch et al.), we allowed for human population growth and age-specific human mortality (i.e. by stratifying the system of ODEs into annual age categories). The number of sandflies per human is assumed to be stable during human population growth and in absence of vector control.

### Parameter quantification

Assumptions about human demography, excess mortality, duration of symptomatic stages of infection, and sandfly biology were based on literature and published data sources (Table 1) [25, 32–39]. Note that for model 3, the duration of PKDL is assumed to be 15 years instead of 5 years (models 1 and 2). Next, for each model we defined four sub-variants in terms of assumptions about the duration of the late recovered stage and age-patterns in exposure to sandfly bites. The duration of the late recovered stage was chosen to be two or five years, which were reasonable values, given that the analytical solution of the system of ODEs at equilibrium showed that all three models could only support the data for durations of the late recovered stage less than seven years (Additional file 1, section 5). With regard to age-patterns in exposure to sandfly bites, we assumed that exposure is either fixed, or increases proportionally with body surface area (i.e. a linear increase in sandfly exposure between age 0 to 20 followed by a constant exposure from age 20 onwards). The latter assumption has also been previously used to model the vector-borne diseases onchocerciasis and lymphatic filariasis [40–42].

**Table 1:** Overview of assumptions and pre-set parameters

Parameters	Value^a^	Source
Human birth rate (per 1000 capita, α_H_)	21 (Indian crude birth rate in 2011)	[32]
Human mortality rate (μ_H_)	Age-dependent (Indian mortality rates in 2011)	[33]
Average duration of late recovered stage (years, 1/ρ_RHC_)	2 or 5	Pre-set
Average duration of symptomatic untreated stage (days, 1/ρ_IHS_)	30 (fitting) and 45 (predicting)	Unpublished data
Average duration of symptomatic treatment 1 (days, 1/ρ_*IHT*1_)	30 (fitting) and 2.5 (predicting)	[34]
Average duration of symptomatic treatment 2 (days, 1/ρ_*IHT*2_)	30 (fitting) and 10 (predicting)	[35]
Average duration of putatively recovered stage (months, 1/ρ_*IHT*_)	21	[36]
Average duration of PKDL (years, 1/ρ_*IHL*_)	5 (models 1 and 2) and 15 (model 3)	Expert opinion (EH and MB)
Infectiveness of symptomatic untreated cases (*p*_*IHS*_)	1.0	Reference value
Infectiveness of patients under treatment 1 and 2 (*p*_*IHT*1_, *p*_*IHT*2_)	0.5	Expert opinion (EH and MB)
Infectiveness of PKDL cases (*p*_*IHL*_)	0.5 (models 1 and 2 only; estimated for model 3)	Expert opinion (EH and MB)
Fraction of untreated symptomatic cases that spontaneously, putatively recover (*f*_*p*_)	0.03	[25]
Excess mortality rate among untreated symptomatic cases (per day, μ_K_)	1/150	Assumption
Excess mortality rate among treated symptomatic cases (per day, μ_*KT*_)	1/150 + 1/600 = 1/120 (fitting) and 1/150 (predicting)	[34, 35]
Fraction of failed first-line treatments (*f*_*F*_)	0.05	[37]
Fraction of putatively recovered cases that develop PKDL (*f*_*L*_)	0.05 (set such that models 1 and 2 predicted a prevalence of PKDL between 4.4 and 7.8 per 10,000 capita in India)	[10, 38]
Average life expectancy of the sandfly (days, 1/μ_*F*_)	14	[39]
Average duration of incubation period in sandflies (days, 1/ρ_*EF*_)	5	[62]
Sandfly biting rate (per day, β)	1/4	[63]
Transmission probability sandfly to human (*p*_*H*_)	1.0^b^	Reference value

The parameter values listed here are the same for all three models and their sub-variants, unless indicated otherwise

^a^Parameter values marked with “fitting” only apply to the KalaNet study setting and were therefore only used when fitting the models to the KalaNet data; related to this, different parameter values were used when predicting the impact of IRS (indicated by “predicting”)

^b^The probability that a susceptible person becomes infected when bitten by an infectious sandfly is assumed to be 1; potential overestimation is compensated by the estimated sandfly density per human

Remaining model parameters (sandflies per human, duration of asymptomatic stages of infection, infectiveness of human stages of infection, and proportion of asymptomatic infections that develop symptoms of VL) were estimated based on data from the KalaNet study, a community-based intervention trial in hyper-endemic clusters in Bihar, India, and in the Terai plains in Nepal [27, 28, 43]. The KalaNet data constitute cross-sectional information on DAT status of 21,204 individuals from three time points spanning two years, and information on incidence of VL during the entire two-year study period. For 668 individuals aged 14 and older, PCR testing was performed as well. Further, a subset of individuals were covered in consecutive cross-sectional surveys, allowing derivation of changes in PCR and DAT status. To quantify our model, we used prevalence of DAT-positivity (titre > 1:800, like Stauch et al. [25]), PCR-positivity, PCR andDAT-positivity, incidence of VL and incidence of PCR-positivity (i.e. a change from PCR-negative to positive between two consecutive years), and the prevalence of *L. donovani* in sandflies in Nepal [43] (which in the model we take to be the proportion of sandflies that is infective, like Stauch et al. [25]). An overview of these data is provided in Table A1-2 in Additional file 1, section 3. In the main analysis, we assume that observed levels of PCR and DAT-positivity adequately reflect prevalences of the corresponding stages of infection in our model. The importance of imperfect test sensitivity and specificity was explored using analytical solutions of the equilibria of the system of ODEs (Additional file 1, section 5). We fitted model parameters to country-specific, population-level data, aggregated over years, villages, age, and sex. Because we used an age-structured model, we could take account of the fact that the PCR data were sampled from a sub-population aged 14 years and older, while data on DAT-positivity and VL incidence were sampled from the whole population (in contrast to Stauch et al. [25], who analyzed the KalaNet data as one homogeneous entity).

Model parameters were fitted in two steps. First, we quantified model parameters with regard to duration of stages of asymptomatic infection, fraction of asymptomatic cases that develop VL, and the number of sandflies per human, conditional on preliminary assumption about infectiveness of human stages of infection (which is only determined by the prevalence of infection in sandflies, and can therefore be solved separately, see Additional file 1). The system of ODEs was solved numerically using the *deSolve* package [44] in R (version 3.2.0) [45], and parameters were estimated within a maximum likelihood framework (ignoring the clustered study design, just like Stauch et al. [25]), using the BFGS algorithm from the *optim* package. Prior to every evaluation of the optimization algorithm we let the model reach equilibrium, assuming that the KalaNet data represent an equilibrium situation. Second, we analytically solved the system of ODEs with regard to infectiveness of human stages of infection and the number of sandflies per human, given data on prevalence of infection in sandflies in Nepal (for approach, see Additional file 1). The proportion of putatively recovered cases that develop PKDL was set to 5 % such that the predicted PKDL prevalence for endemic villages in Nepal in models 1 and 2 was 5 per 10,000 population, which corresponds to the 4.4 to 7.8 per 10,000 that has been reported for Nepal [10]. Last, for each model we selected the best sub-variant based on the log-likelihood with regard to age-patterns in prevalence of infection markers and incidence of VL and PCR-positivity.

### Predicting the impact of IRS

With each best sub-variant of model 1, 2, and 3, we simulated a high, medium, and low endemic setting, defined in terms of pre-IRS VL incidence of 20 per 10,000, 10 per 10,000 and 5 per 10,000 per year, respectively. These endemic settings were chosen given the declining trends in VL cases and the fact that VL incidences of 20 cases per 10,000 capita per year (as observed in the KalaNet setting) are currently rarely observed [46, 47]. Each endemic setting was quantified by tuning the number of sandflies per human, assuming that transmission dynamics are in equilibrium with current detection and treatment interventions (which are slightly different from those in the KalaNet situation; see Table 1). We simulated the impact of IRS strategies as planned for India, i.e. two spraying rounds per year targeting houses and cattle sheds in endemic villages [18]. We assumed that optimally implemented IRS (*optimal IRS*) results in a continuous reduction in sandfly density of approximately 63 %, given the reported reduction in sandfly density after IRS with dichlorodiphenyltrichloroethane (DDT) of 72 % [48] and the assumption that rotating spraying teams continuously cover households 85 %-95 % of the time. Sub-optimally implemented IRS (*sub-optimal IRS*) was assumed to be half as effective due to lower continuous household coverage, sub-optimal spraying techniques and sandfly resistance to DDT [19–23], leading to a continuous sandfly density reduction of 31.5 %. We interpreted the WHO elimination target in our model as an annual incidence of VL cases (receiving treatment) of <1 per 10,000 capita.

In a sensitivity analysis for predicted trends in VL incidence during IRS, we varied the values of key estimated and assumed parameter values by factors 4/5 and 5/4 (except for the number of sandflies per human, as this parameter mainly influences predicted trends in VL incidence through pre-IRS infection levels).

## Results

All four sub-variants of all three models could closely reproduce the country-specific, population-level incidence and prevalence data, with deviances ranging between 2.11 and 2.61 9 (*χ*^2^ degrees of freedom = 8, *p* > > 0.5). All model sub-variants estimated the duration of early asymptomatic infection (PCR+/DAT-) at around 1.1 years and the duration of late asymptomatic infection (PCR+/DAT+, excluding cases with symptoms) at just under four months. Estimates for the proportion of asymptomatically infected cases that develop VL (range 2.8–3.9 %), infectiveness of early and late asymptomatic infection (0.014–0.018 and 0.027–0.035, respectively, model 1 and 2 only), infectiveness of PKDL (2.32–2.72, model 3 only), and duration of the early recovered stage (1.0 to 1.7 years; PCR-/DAT+, excluding putatively recovered people) slightly varied between models and sub-variants (i.e. assumptions about age-dependent exposure to sandfly bites and duration of the late recovered stage). All fitted parameter values are presented in Table 2.

**Table 2:**
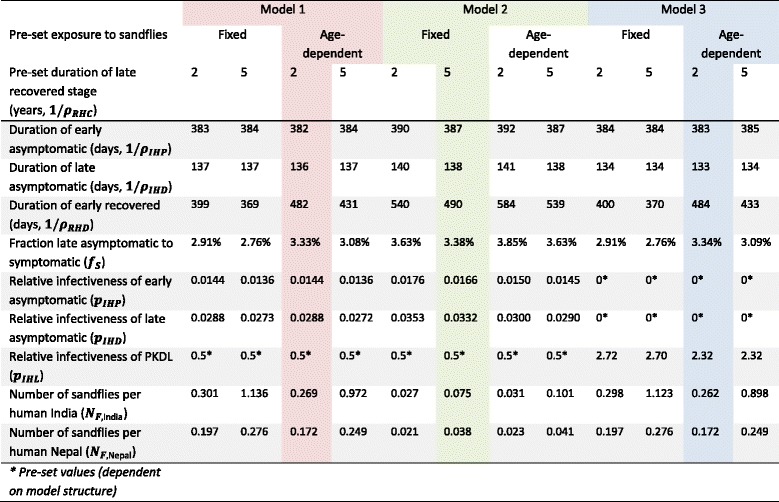
Quantified parameter values of the twelve model variants

**Fig. 2:**
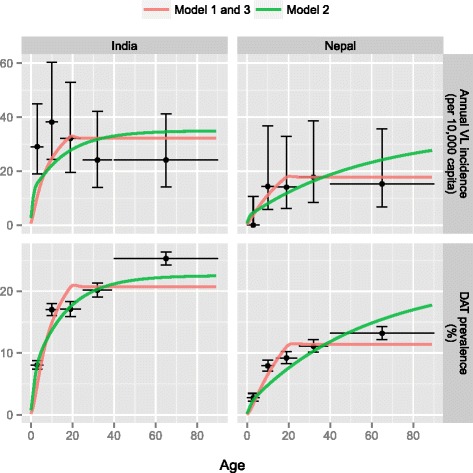
Predicted and observed age-patterns in VL incidence and DAT prevalence in India and Nepal. Coloured lines represent model predictions from the sub-variant of each of the three models that best fit age-patterns in human infection markers; black bullets represent the data per age group; horizontal lines indicate the age range for each data point; vertical lines represent 95 %-Bayesian credible intervals, given total raw sample sizes (i.e. not accounting for clustering, see Additional file 1 for sample sizes). See Additional file 2 for illustrations of the fit of all model sub-variants to all data types

The colours represent the model sub-variants that best reproduced the age-structured prevalence and incidence data. See Additional file 2 for illustrations of fitting of all model variants to all data and Fig. 2 for the predicted and observed age-patterns in VL incidence and DAT prevalence in India and Nepal with the selected model variants

Given the parameter estimates above, the most common infection history for a person to go through (susceptible, asymptomatically infected, and early recovered without ever developing VL) takes on average about 2.7 to 3.1 years (not including the duration of the late recovered stage, which we assume to be either two or five years). This is in line with the observation that only 6 out of 668 subjects who were tested with PCR were positive in year 1, negative in year 2, and again positive in year 3. All three models predicted that in a state of endemic equilibrium about 10 % of all transmission of infection is generated by VL cases (treated and untreated). According to models 1 and 2, an additional 8 % of transmission is generated by PKDL cases and the remaining 82 % by asymptomatically infected cases. In model 3, 90 % of transmission is generated by PKDL cases (and none by asymptomatic infections, by default).

The sub-variants of models 1 and 3 that best reproduced the age-specific data were based on the assumptions of age-dependent exposure to sandflies and a duration of late recovered stage of two years; for model 2, the sub-variant with fixed exposure to sandflies and duration of the late recovered stage of five years best fitted the data. Figure 2 illustrates the fit of the best sub-variants to the age-specific data on VL incidence and DAT prevalence, with identical fits for model 1 and 3. Fits to other data types (PCR incidence, PCR prevalence, PCR/DAT prevalence) and fits for all model sub-variants can be found in Additional file 2.

**Fig. 3:**
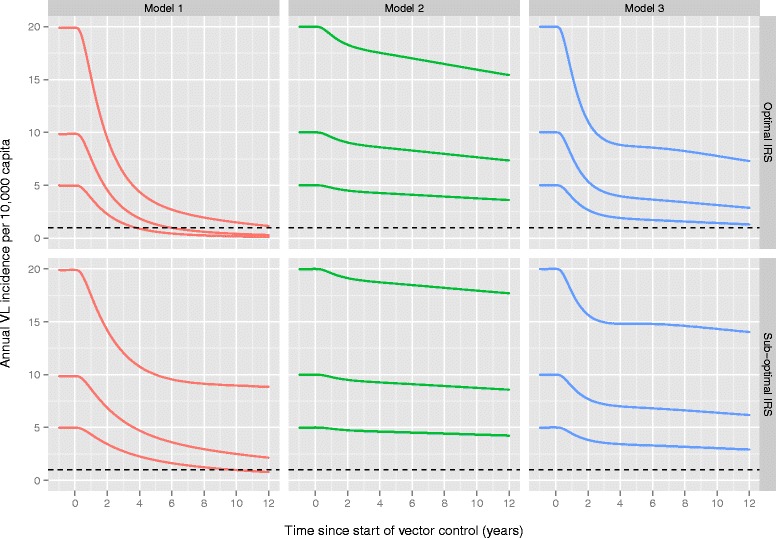
Predicted impact of optimal and sub-optimal IRS on VL incidence for three endemic settings. IRS is assumed to start in the year zero. Lines within plots represent different pre-IRS endemic settings (high: 20/10,000, medium: 10/10,000, low: 5/10,000); the dotted line represents the target VL incidence of <1 per 10,000 capita. Model predictions were made with the sub-variant of each of the three models that best fit age-patterns in human infection markers. See Additional file 3 for the short and long-term impact of optimal and sub-optimal IRS in low, medium, and highly endemic settings with all model sub-variants

Using the best sub-variant of each model, we predicted the impact of optimal and sub-optimal IRS on VL incidence for high, medium and low endemic settings (Fig. 3). Models 1 and 3 predict that optimal IRS (63 % assumed reduction in sandfly density) reduces VL incidence by about 25 % in the first year and by another 25 % of the original incidence level in the second year after the start of IRS, irrespective of the endemicity level at equilibrium. However after two years, the predictions of model 1 and 3 diverge: in model 1, VL incidence keeps on declining due to the rapid depletion of the reservoir of infection in asymptomatically infected cases (average duration of asymptomatic infection of about 1.4 years); in model 3, the reduction in VL incidence slows down strongly after two years due to the presence of the relatively large reservoir of infection in PKDL-cases (average duration of 15 years). Model 2 predicts a relatively slow and stable decline from the start of IRS, as the decrease in sandfly density is assumed to have no influence on VL cases arising from people in whom old infection reactivates.

Model 1 predicts that about 4 to 6 years of optimal IRS will reduce the annual VL incidence in low and medium endemic settings to levels (just) under 1 per 10,000 capita. However, models 2 and 3 predict that these low levels of VL incidence cannot even be achieved within 12 years of optimal IRS. Similarly, model 1 predicts that with sub-optimal IRS, these levels of VL incidence are only achieved after about 10 years, and only in low endemic settings. Still, when IRS is continued over an extremely long period of time (say 200 years), most sub-variants of the three models predict that optimal IRS will eventually result in elimination in all endemic settings (Additional file 3). Sub-optimal IRS will only lead to reaching the target in low and medium endemic settings, with varying durations of IRS required per model. Additional file 3 also illustrates that for model 1 (and 3 to a lesser extend), the predictions depend on the duration of the late recovered stage in high endemic settings and with sub-optimal IRS: longer (5 year) duration leads to a slower decline in VL incidence, and a faster re-occurrence of infection. For model 2, the duration of the late recovered stage on the impact of IRS is negligible. For model 3, the deceleration of the decline in VL incidence is largely a function of the duration of PKDL. A longer duration of PKDL will generate a longer infection pressure towards the sandfly and therefore slow down the decreasing VL incidence.

**Fig. 4:**
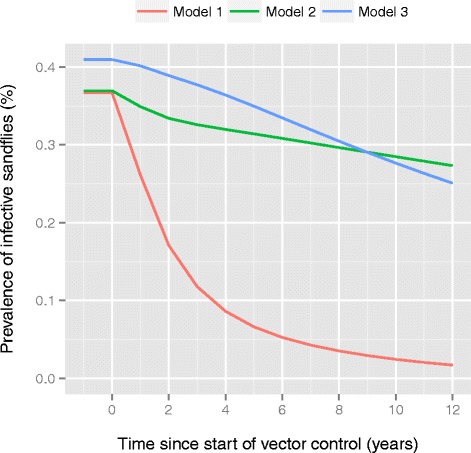
Predicted prevalence of infective sandflies during IRS. Pre-IRS prevalence levels of infective sandflies represent a setting with 10 annual VL cases per 10,000 capita. IRS is assumed to start in the year zero, and to be implemented optimally (63 % reduction in sandfly density). The three colored lines represent the sub-variant of each of the three models that best fit age-patterns in human infection markers. See Additional file 4 for low, medium and highly endemic settings with optimal and sub-optimal IRS

Figure 4 illustrates trends in prevalence of infective sandflies (among caught sandflies) for a medium endemic setting with optimal IRS (see Additional file 4 for low and highly endemic settings). Compared to model 1, models 2 and 3 predict a relatively slow decline in prevalence of infective sandflies because of the persisting parasitic reservoirs of late recovered and PKDL cases, respectively.

Additional file 5 provides an overview of the results of the sensitivity analysis for a medium endemic setting with optimal IRS. Only the assumed effect of IRS (high and low values were 5/4 and 4/5 of the value used in the main analysis) directly influenced predicted trends without changing pre-control infection levels. The duration of IRS required to achieve the elimination target (only relevant in model 1) was most sensitive for the parameter values of the effect of IRS (4 and 9 years until elimination), the duration of the early asymptomatic stage of infection (4 and 8.5 years until elimination), and the proportion of infections that result in symptoms (4.5 and 8 years until elimination). Sensitivity of predicted trends in VL incidence during IRS were strongly associated with changes in pre-control infection levels (i.e. alternative parameter values often produced parallel trends in VL incidence). The predictions by model 3 were most sensitive to the proportion of individuals developing symptoms and PKDL, and the infectiveness and duration of PKDL (illustrated in Additional file 5). The transmission dynamics are insensitive to the assumed infectiveness of early asymptomatic cases relative to late asymptomatic cases (data not shown).

## Discussion

We developed three structurally different models with different reservoirs of infection to predict the impact of IRS on VL incidence on the ISC, using the KalaNet dataset from India and Nepal to quantify transmission dynamics in each model. All three models could explain the KalaNet data equally well. However, the predicted impact of IRS varied substantially between models, such that a conclusion about reaching the VL elimination targets for the ISC heavily depends on assumptions about the main reservoir of infection in humans: asymptomatic cases (model 1), recovered (immune) individuals in whom infection reactivates (model 2), or PKDL cases (model 3). Biologically, a mixture of the different models is most likely, but could not be quantified solely based on the KalaNet data. Still, given that the three models predict markedly different trends of VL incidence and infection in sandflies during IRS, we may be able to express preference for one of the models based on field data regarding the impact of IRS.

So far, only a limited amount of field data on the impact of IRS on VL incidence has been published [49]. Kumar et al. report that after one year of active IRS in 19 districts of Bihar, VL incidence decreased by 49–100 % in 15 districts, and VL incidence was stable or even increased in 4 districts, such that the average reduction in VL prevalence over all 19 districts was about 50 %. Based on these findings we tentatively conclude that the models with the infection reservoir in asymptomatic cases (model 1) and PKDL cases (model 3) are probably closer to reality than the model with the disease reservoir in re-activating recovered cases (model 2). Although there is literature on prevalence of infection in sandflies [43, 50, 51] and the impact of IRS on sandfly density [20, 21, 52], unfortunately, there are no published data on the impact of IRS on prevalence of infection in sandflies. Such data would be very valuable to further our understanding of VL transmission dynamics, and distinguish between model 1 and 3 the model that is closest to reality. Still, as model 3 was included as an extreme variant of model 1, we consider model 1 to be the most realistic of our set of models. Currently ongoing initiatives such as the CARE project, that is taking place in Bihar India, [53] are anticipated to provide more data on the long-term impact of IRS on VL incidence and perhaps prevalence of infected sandflies in the field, which will be crucial to validate model predictions and better understand VL transmission dynamics.

The large scale implementation of IRS with DDT in India started in 2005 as part of the national VL elimination program [54], twelve years before the targeted year of VL elimination, 2017. Assuming that model 1 is closest to reality, elimination of VL (incidence <1 per 10,000 capita) is feasible in low, medium and highly endemic settings by means of about four, six and twelve years of optimal IRS, respectively. With sub-optimal IRS, which in some settings may still be too optimistic, model 1 predicts that the elimination target can only be achieved in low endemic settings within about 10 years. Assuming that in some highly endemic areas IRS was only implemented after the release of the WHO NTD Roadmap and London Declaration in 2012, IRS would have to reduce sandfly densities by at least about 85 % to achieve the elimination target in the following 5 years (by 2017). With our assumed 63 % reduction in sandfly density by optimal IRS, the elimination target can be achieved within 5 years (i.e. by 2017 if IRS was only implemented in 2012) for settings with an annual VL incidence of up to about 8 per 10,000 capita. The outlook would be much poorer if IRS actually has been implemented sub-optimally. In particular for areas with highly endemic levels, a longer period and/or higher effectiveness of IRS will be required, ideally supplemented by additional interventions, certainly if the level of IRS is sub-optimal. DDT is interpreted to have an insecticidal effect on the sandfly; an insect-repellent effect would have led to a decreased biting rate, with a relatively lower impact on the transmission and VL incidence In the future, the use of DDT is expected to be phased out and replaced by synthetic pyrethroids, due to the increasing sandfly resistance to DDT [23] and its negative environmental impact [55]. In the further future, vaccination may be an important additional tool to eliminate VL on the ISC, should a vaccine become available [56, 57]. Our models provide a tool to explore the potentional impact of future vaccines and identify the target product profiles of vaccines that may achieve the elimination target.

Our study is based on the existing deterministic transmission model that was developed at Tuebingen University by Stauch et al. [25], but we considerably improved the model in several ways. To better account for the human demography on the ISC, we added population growth and age-specific mortality. The resulting age-structured model further allowed us to better mimic age-patterns in the KalaNet data. This also allowed us to account for the fact that the PCR data in the KalaNet study were collected from a subsample of individuals aged 14 and older. Unlike Stauch et al., we purposely did not use data on leishmanin skin testing (LST, which was associated with the late recovered, immune stage), as these LST data did not originate from the same study area. Moreover, the fraction LST positive used and the assumption that early asymptomatic infection (PCR+/DAT-) lasts only 60 days (we estimate 1.1. year) caused the original model to predict a very short natural history of infection; one cycle of asymptomatic infection, recovery, and loss of immunity was predicted to only take about 450 days, on average. Instead, we chose plausible values for the duration of the recovered, immune stage (two or five years, which could readily support the data as shown by the solutions to the system of ODEs in equilibrium), and used data on PCR incidence and prevalence of PCR and DAT-positivity to inform the model about the duration of the natural history of asymptomatic infection. We further improved the model by fitting our models to country-specific data (India vs. Nepal), and by taking account of the fact that the data on prevalence of infection in sandflies was only collected in Nepal.

Although our model was based on detailed field data, several uncertain factors remained. We interpreted the KalaNet dataset as if it represented an endemic equilibrium. However, in reality repeating small outbreaks of symptomatic cases have been reported to occur [58]. Whether these fluctuations are true outbreaks or simple stochastic variation remains to be clarified, which will require more modelling and detailed longitudinal data. We will investigate this in the future, using an individual-based model (based on the current study) that captures both stochastic and spatial variation. In our analyses, we assume that the KalaNet data represent an endemic equilibrium, which is reasonable given the slow transmission dynamics in all three models; this slowness is not a result of the equilibrium assumption, but due to the large and stable reservoir of infection in asymptomatic individuals (model 1), reactivating past infections (model 2), or PKDL cases (model 3). The KalaNet study included an active case-finding strategy, and although we accounted for a longer duration of the symptomatic untreated stage for our predictions, 45 instead of 30 days, the time between onset of symptoms and treatment could in certain settings be longer. This resulted in an increase in the number of predicted deaths due to VL but hardly influenced the transmission dynamics or the predicted duration until reaching the elimination target. Another potential limitation of our study is that observed levels of PCR and DAT-positivity were assumed to adequately reflect the prevalences of the corresponding stages of infection in the model. In a meta-analysis, Chappuis et al. found that sensitivity and specificity of DAT testing for the diagnosis of VL were fairly high (about 97.1 % and 95.7 % respectively)" [59], but these estimates do not necessarily apply to the ascertainment of *L.donovani* asymptomatic infection, as the DAT test was not validated as such for that purpose. Further, we interpreted the DAT data at the 1:800 titre cut-off (instead of the standard cut-off of 1:1600), which probably increased test sensitivity but decreased specificity. There is little information regarding the sensitivity and specificity of PCR, as there is no gold standard [60]. An exploratory analysis of accounting for imperfect DAT and PCR testing in fitting the KalaNet data showed that predictions for the impact of IRS only vary marginally when using realistic values of sensitivity and specificity (Additional file 1, section 5). Further, the duration of the early asymptomatic stage suggests that the development of detectable antibodies after infection requires about 1 year, which seems relatively long. However, the estimated duration of the early asymptomatic stage was only at most 7 % lower when sensitivity of PCR testing was assumed to be as low as 70 %. This can be explained by the fact that PCR sensitivity affects PCR prevalence and incidence in the same way (although the effect on incidence is somewhat larger due to the involvement of two measurements). Our estimate of the duration of immunity after clearance of infection (approximately 3 years, of which two year were assumed to be spent in a DAT-negative state), is very similar to that by Chapman et al. [61], who recently analysed rK39 and LST data from Bangladesh using a Markov model. There are differences in the estimates of the duration of the of asymptomatic stage: 5 months (Chapman et al.) and 1.5 years in this study, and the percentage of asymptomatic individuals that develop clinical symptoms: 14.7 % (Chapman et al.) and 3.3 % in this study. These differences may be well explained by differences in the type of data (geographic region and type of diagnostic tests) and modelling methods used (the use of a full transmission model is the strength of the current study). Lastly, we could only estimate infectiveness of human stages of infection indirectly from the prevalence of infection in sandflies, and only after certain assumptions about the relative infectiveness of clinical cases. Ongoing xenodiagnostic studies and additional longitudinal data on the prevalence of infection in sandflies during interventions are anticipated to further inform the model regarding this aspect.

## Conclusions

We conclude that several structurally different models can explain population-level data on VL transmission equally well. Consequently, the predicted impact of IRS strongly depends on assumptions about the reservoir of infection in humans. Data on the impact of IRS available so far suggest one model is probably closest to reality (model 1, where asymptomatic individuals represent the main reservoir of infection). According to this model, elimination of VL (incidence of <1 per 10,000 capita) is probably only feasible by 2017 in low and medium endemic settings with optimal IRS; in highly endemic settings and settings with sub-optimal IRS, additional interventions will be required.

## Additional files

Additional file 1:
**This document provides the description, characterisation and calculations of equilibria of a system of ordinary differential equations for three VL transmission models along with data.** (DOCX 1704 kb) Additional file 2:
**Supplementary figure illustrating the fit of all model sub-variants to all data types (extended version of Fig.** 2 **in the main manuscript).** (PDF 95 kb) Additional file 3:
**Supplementary figure illustrating the long-term and short-term impact of optimal and sub-optimal IRS in low, medium, and highly endemic areas for all model variants (extended version of Fig.** 3 **in the main manuscript), as well as transmission dynamics when infection is introduced in a susceptible human population (outbreak).** (PDF 7486 kb) Additional file 4:
**Supplementary figure illustrating the impact of optimal IRS on the prevalence of infective sandflies in low, medium, and highly endemic settings, according to the best sub-variant of each model (extended version of Fig.** 4 **in the main manuscript).** (PDF 7 kb) Additional file 5:
**Supplementary figure illustrating the impact of optimal IRS on incidence of VL in a sensitivity analysis of key estimated and assumed parameter values.** (PDF 559 kb)

## Abbreviations

DAT: Direct agglutination test
IRS: Indoor residual spraying
ISC: Indian subcontinent
KA: Kala-azar
LST: Leishmanin skin test
NTD: Neglected tropical disease
ODE: Ordinary differential equation
PCR: Polymerase chain reaction
PKDL: Post-kala-azar dermal leishmaniasis
VL: Visceral leishmaniasis
WHO: World Health Organization
