# Supplementary material for: Feasibility of eliminating visceral leishmaniasis from the Indian subcontinent: explorations with a set of deterministic age-structured transmission models
Source: Parasit Vectors. 2016 Jan 19;9:24. doi: 10.1186/s13071-016-1292-0 (PMC4717541; doi:10.1186/s13071-016-1292-0)
Supplement: Additional file 1: — This document provides the description, characterisation and calculations of equilibria of a system of ordinary differential equations for three VL transmission models along with data. (DOCX 1704 kb) [file 13071_2016_1292_MOESM1_ESM.docx]

**ADDITIONAL FILE 1**

**Description, characterisation, and calculations of equilibria for a system of ordinary differential equations for a set of VL transmission models**

Epke A le Rutte^1*^, Luc E Coffeng^1^, Daniel M Bontje^1^, Epco C Hasker^2^, José A Ruiz Postigo^3^, Daniel Argaw^3^, Marleen C Boelaert^2^, Sake J de Vlas^1^.

*Corresponding author: [e.lerutte@erasmusmc.nl](mailto:e.lerutte@erasmusmc.nl)

^1^ Department of Public Health, Erasmus MC, University Medical Center Rotterdam, PO Box 2040, 3000 CA Rotterdam, the Netherlands
^2^ Institute of Tropical Medicine, Nationalestraat 155, 2000, Antwerp, Belgium
^3^ World Health Organization, Geneva, Switzerland

# Table of contents

Table of contents 2

1 Model structure 3

2 System of ordinary differential equations 7

3 Data 9

4 Derivation of equations for the system of ODEs at equilibrium 10

4.1 First-line treatment of symptomatic cases (PCR+/DAT+) 11

4.2 Untreated symptomatic cases originating from late asymptomatic infection (PCR+/DAT+) 11

4.3 Second-line treatment of symptomatic cases (PCR+/DAT+) 12

4.4 Putatively recovered (PCR-/DAT+) 12

4.5 Post kala-azar dermal leishmaniasis (PCR+/DAT+) 12

4.6 Early asymptomatic (PCR+/DAT-) 13

4.7 Late asymptomatic (PCR+/DAT+) 13

4.8 Fraction of late asymptomatic cases that develop clinical disease 13

4.9 Early recovered (PCR-/DAT+) 14

4.10 Late recovered (PCR-/DAT-) 15

4.11 Susceptible humans (PCR-/DAT-) 15

4.12 Force of infection acting on humans and number of flies per human 16

4.13 Number of flies per human 16

4.14 Infectivity of human infected stages towards the sandfly 17

5 R calculations for equilibrium state of system of ODEs 19

5.1 Scenario analysis for reservoir of infection in symptomatic cases only 23

5.2 Scenario analysis for imperfect DAT testing 24

5.3 Scenario analysis for imperfect PCR testing 24

6 References 27

# Model structure

Figures A1-1, A1-2, and A1-3 provide schematic representations of three model structures, which are adapted from a model developed by Stauch *et al* [1]. Where possible we used the same notation as Stauch *et al*, where $H$ stands for human and $F$for sandfly. Susceptible populations are denoted by $S$, latent infections by $E$, infective cases by $I$, and recovered cases by $R$. For instance, $S_{F}$, $E_{F}$ and $I_{F}$ represent the susceptible, latent infected, and infective sandfly states, respectively. Most human states have an extended subscript, for example $I_{HT1}$, which represents infective cases under first-line treatment. Table A1-1 provides an overview of all model compartments and parameters, and the symbols by which there are represented.

Figure A1-1. Schematic representation of the structure of Model 1. In this model, cases of early and late asymptomatic infection (compartments $\boldsymbol{I}_{\boldsymbol{HP}}$ and $\boldsymbol{I}_{\boldsymbol{HD}}$, respectively) together constitute the main reservoir of infection that sustains transmission. Fully recovered DAT-negative people ($\boldsymbol{R}_{\boldsymbol{HC}}$) may lose immunity (i.e. transition from $\boldsymbol{R}_{\boldsymbol{HC}}$ to $\boldsymbol{S}_{\boldsymbol{H}}$) and be reinfected.


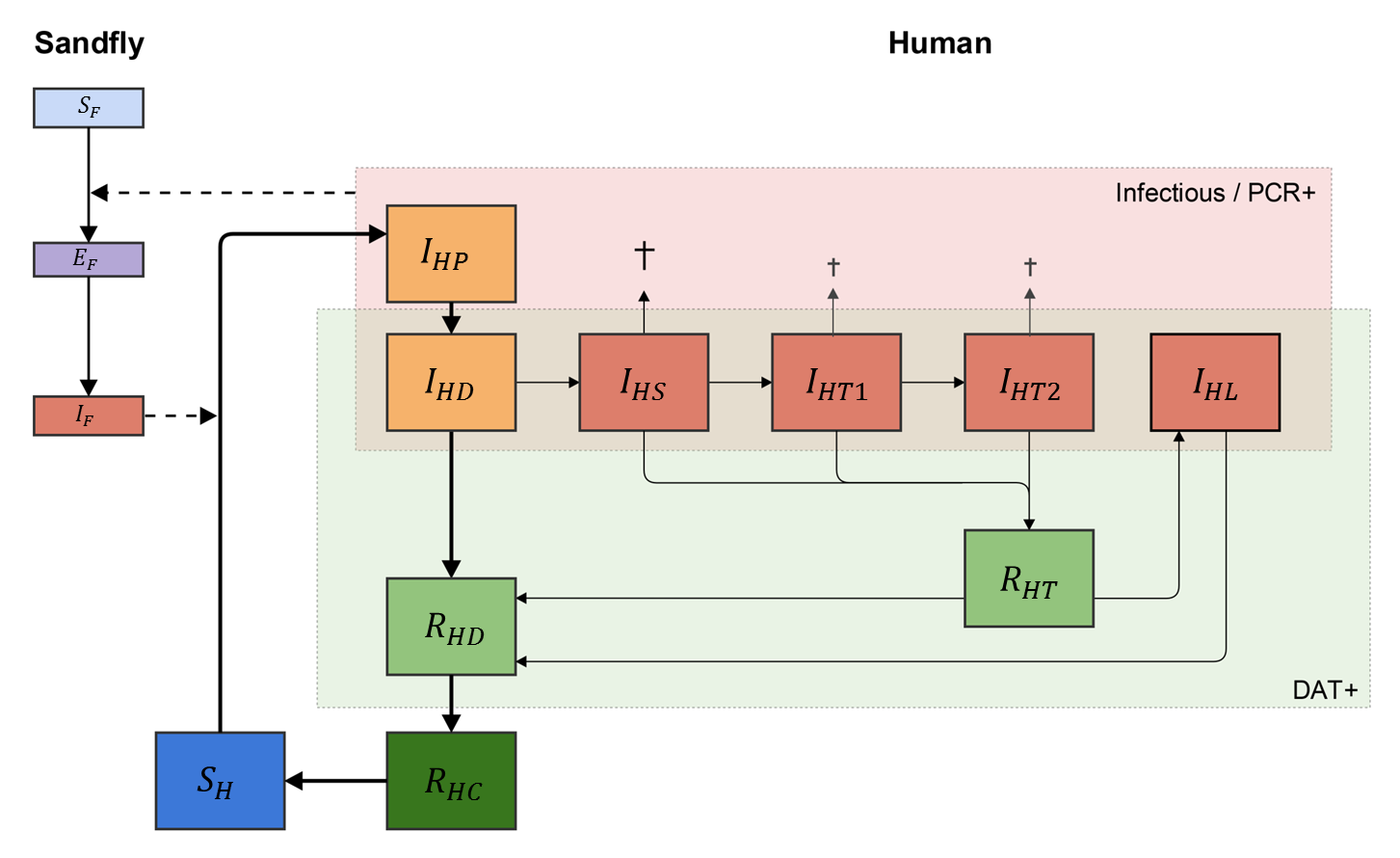


Figure A1-2. Schematic representation of the structure of Model 2. In this model, cases of early and late asymptomatic infection (compartments $\boldsymbol{I}_{\boldsymbol{HP}}$ and $\boldsymbol{I}_{\boldsymbol{HD}}$, respectively) together constitute the main reservoir of infection that sustains transmission. Further, fully recovered DAT-negative people ($\boldsymbol{R}_{\boldsymbol{HC}}$) do no lose immunity (model 1), but instead their infection may reactivate after a certain period (i.e. transition from $\boldsymbol{R}_{\boldsymbol{HC}}$ to $\boldsymbol{I}_{\boldsymbol{HP}}$).


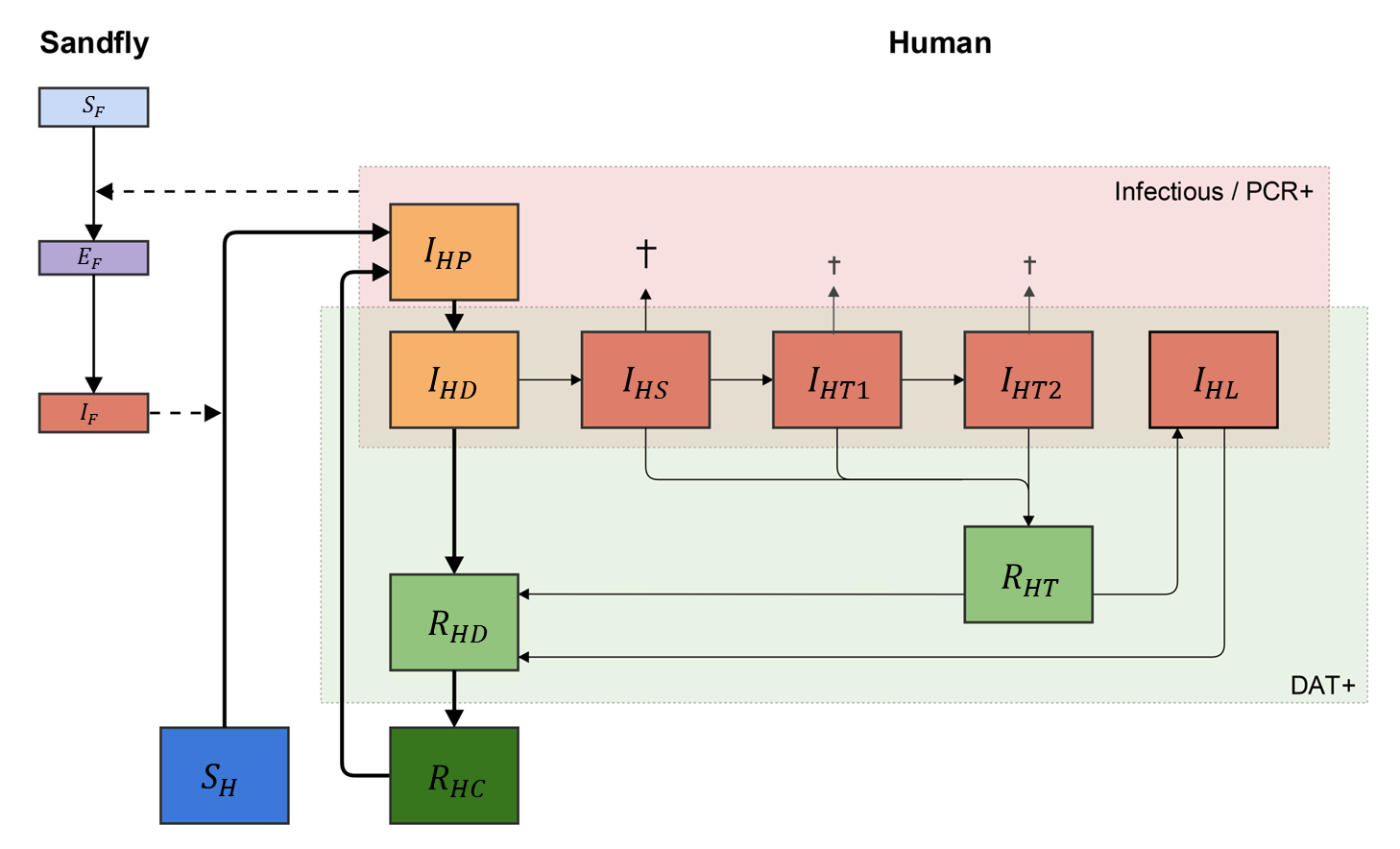


Figure A1-3. Schematic representation of the structure of Model 3. In this model, cases of post kala-azar dermal leishmaniasis (PKDL, compartment $\boldsymbol{I}_{\boldsymbol{HL}}$) constitute the reservoir of infection that sustains transmission of infection. In other words, asymptomatically infected people ($\boldsymbol{I}_{\boldsymbol{HP}}$ and $\boldsymbol{I}_{\boldsymbol{HD}}$) do not contribute to transmission of infection. As in model 1, fully recovered DAT-negative people ($\boldsymbol{R}_{\boldsymbol{HC}}$) may lose immunity (i.e. transition from $\boldsymbol{R}_{\boldsymbol{HC}}$ to $\boldsymbol{S}_{\boldsymbol{H}}$) and be reinfected.


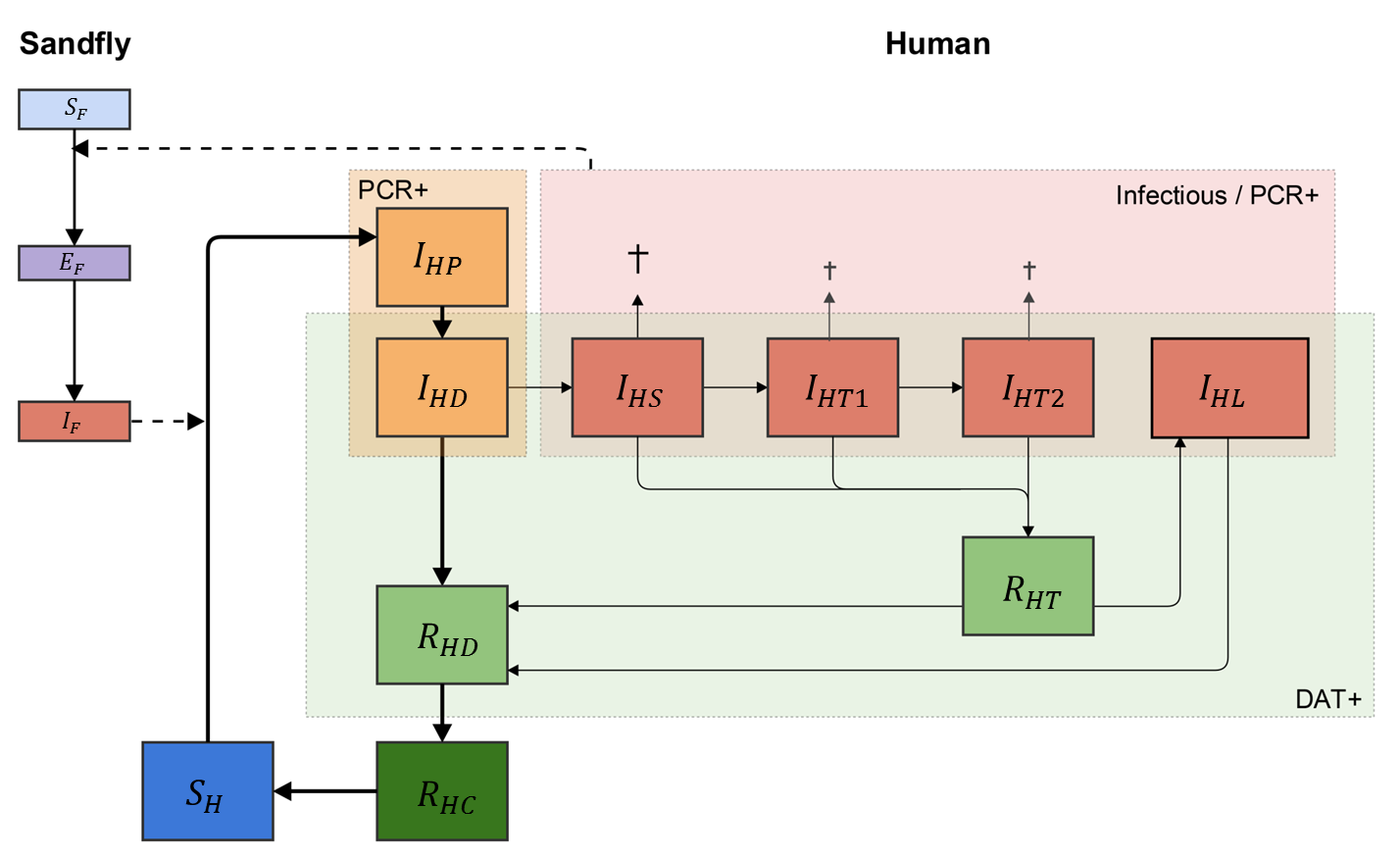


Table A1-1. Overview of model compartments and parameters, and their interpretation.

| Symbol | Interpretation* |
| --- | --- |
| $N_{H}$ | Size of human population |
| $S_{H}$ | Susceptible humans (PCR- / DAT -) |
| $I_{HP}$ | Humans with early asymptomatic infection (PCR+ / DAT-) |
| $I_{HD}$ | Humans with late asymptomatic infection (PCR+ / DAT+) |
| $I_{HS}$ | Humans with symptoms of infection (kala-azar; PCR+ / DAT+) |
| $I_{HT1}$ | Humans under first-line treatment (PCR+ / DAT+) |
| $I_{HT2}$ | Humans under second-line treatment (PCR+ / DAT+) |
| $R_{HT}$ | Humans that are putatively recovered after treatment (PCR- / DAT+) |
| $I_{HL}$ | Humans with post kala-azar dermal leishmaniasis (PKDL; PCR+ / DAT+) |
| $R_{HD}$ | Early recovered humans (PCR- / DAT+) |
| $R_{HC}$ | Late recovered humans (PCR - / DAT-) |
| $\boldsymbol{\rho}_{\boldsymbol{X}}$ | Rate at which humans (or flies) leave compartment $\boldsymbol{X}$ (i.e. 1 / average duration of state $\boldsymbol{X}$**)** |
| $\alpha_{H}$ | Human birth rate per capita (whole population) |
| $\mu_{H}$ | Human background mortality rate |
| $\mu_{KA\text{, untreated}}$ | Excess mortality among humans with untreated symptomatic infection |
| $\mu_{KA\text{, treated}}$ | Excess mortality among humans with treated symptomatic infection |
| $f_{S}$ | Proportion of humans with late asymptomatic infection that develop clinical symptoms |
| $f_{P}$ | Proportion of humans with clinical symptoms that recover without treatment |
| $f_{F}$ | Proportion of humans with clinical symptoms in whom first-line treatment fails |
| $f_{L}$ | Proportion of humans with clinical symptoms that develop post kala-azar dermal leishmaniasis |
| $f_{A}$ | Proportion of late recovered humans in whom infection reactivates |
| $N_{F}$ | Number of sandflies per human |
| $S_{F}$ | Susceptible sandflies |
| $E_{F}$ | Exposed / latent infected sandflies |
| $I_{F}$ | Infective sandflies |
| $\mu_{F}$ | Mortality and birth rate of sandflies (assuming that the number of sandflies per human is stable) |
| $\lambda_{F}$ | Force of infection acting on flies |
| $\lambda_{H}$ | Force of infection acting on humans |
| $\beta$ | Sandfly biting rate (1 / average time period between consecutive bites) |
| $p_{X}$ | Infectivity of human state $X$ towards sandflies |
| $p_{H}$ | Probability that an infective sandfly transmits infection to a human during a blood meal |
| * PCR: polymerase chain reaction for detection of parasite DNA; DAT: direct agglutination test for detection of antibodies against *Leishmania donovani*. | |

# System of ordinary differential equations

Below, we describe the system of ordinary differential equations (ODEs) for models 1, 2, and 3. Models 1 and 2 are distinguished by the value of $f_{A}$ (model 1: $f_{A}=0$; model 2: $f_{A}=1$). In model 3 ($f_{A}=0$), the infectivity of asymptomatic cases infections is set to zero, such that PKDL is the main reservoir of infection. Other than that, the three models can be described with exactly the same parameters. Figure A1-4 provides a schematic representation of the common structure of all three models, along with parameter and compartment symbols.

Human compartments, defined in terms of absolute human population size:

$$\frac{dS_{H}}{dt}= N_{H}\cdot\alpha_{H}+\left( 1-f_{A} \right)\cdot\rho_{RHC}\cdot R_{HC}-\left( \lambda_{H}+\mu_{H} \right)\cdot S_{H}$$

$$\frac{dI_{HP}}{dt}=\lambda_{H}\cdot S_{H}+f_{A}\cdot\rho_{RHC}\cdot R_{HC}-\left( \rho_{IHP}+\mu_{H} \right)\cdot I_{HP}$$

$$\frac{dI_{HD}}{dt}=\rho_{IHP}\cdot I_{HP}-\left( \rho_{IHD}+\mu_{H} \right)\cdot I_{HD}$$

$$\frac{dI_{HS}}{dt}=f_{S}\cdot\rho_{IHD}\cdot I_{HD}-\left( \rho_{IHS}+\mu_{H}+\mu_{KA\text{, untreated}} \right)\cdot I_{HS}$$

$$\frac{dI_{HT1}}{dt}=\left( 1-f_{P} \right)\cdot\rho_{IHS}\cdot I_{HS}-\left( \rho_{IHT1}+\mu_{H}+\mu_{KA\text{, treated}} \right)\cdot I_{HT1}$$

$$\frac{dI_{HT2}}{dt}=f_{F}\cdot\rho_{IHT1}\cdot I_{HT1}-\left( \rho_{IHT2}+\mu_{H}+\mu_{KA\text{, treated}} \right)\cdot I_{HT2}$$

$$\frac{dR_{HT}}{dt}=f_{P}\cdot\rho_{IHS}\cdot I_{HS}+\left( 1-f_{F} \right)\cdot\rho_{IHT1}\cdot I_{HT1}+\rho_{IHT2}\cdot I_{HT2}-\left( \rho_{RHT}+\mu_{H} \right)\cdot R_{HT}$$

$$\frac{dI_{HL}}{dt}=f_{L}\cdot\rho_{RHT}\cdot R_{HT}-\left( \rho_{IHL}+\mu_{H} \right)\cdot I_{HL}$$

$$\frac{dR_{HD}}{dt}=\left( 1-f_{S} \right)\cdot\rho_{IHD}\cdot I_{HD}+\left( 1-f_{L} \right)\cdot\rho_{RHT}\cdot R_{HT}+\rho_{IHL}\cdot I_{HL}-\left( \rho_{RHD}+\mu_{H} \right)\cdot R_{HD}$$

$$\frac{dR_{HC}}{dt}=\rho_{RHD}\cdot R_{HD}-\left( \rho_{RHC}+\mu_{H} \right)\cdot R_{HC}$$

Fly compartments, defined in terms of flies per human $N_{F}$, assuming that the fly population grows proportionally with the human population size $N_{H}$ (i.e. $N_{F}$ remains constant in absence of vector control, and the sandfly hatching rate is equal to the total sandfly mortality rate):

$$\frac{dS_{F}}{dt}=\mu_{F}\cdot N_{F}\cdot\left( 1-\text{effect}_{\text{IRS}} \right)-\left( \lambda_{F}+\mu_{F} \right)\cdot S_{F}$$

$$\frac{dE_{F}}{dt}=\lambda_{F}\cdot S_{F}-\left( \rho_{EF}+\mu_{F} \right)\cdot E_{F}$$

$$\frac{dI_{F}}{dt}=\rho_{EF}\cdot E_{F}-\mu_{F}\cdot I_{F}$$

Force of infection acting on humans, assuming that biting rate $\beta$ is independent of the size of the human and sandfly populations:

$$\lambda_{H}=\beta\cdot p_{H}\cdot I_{F}$$

Force of infection acting on flies, assuming that biting rate $\beta$ is independent of the size of the human and sandfly populations:

$$\lambda_{F}=\beta\cdot\frac{\sum_{X=I_{HP},\text{ }I_{HD},\text{ }I_{HS},\text{ }I_{HT1},\text{ }I_{HT2},\text{ }I_{HL}} X\cdot p_{X}}{N_{H}}$$

As mentioned above, in model 3, $p_{IHP}=p_{IHD}=0$, such that only clinical human cases contribute to transmission of infection. Further, in model 3 we set the duration of PKDL ($1/\rho_{IHL}$) thrice as long as in model 1 and 2, such that PKDL is the main reservoir of infection.

Figure A1-4. Schematic representation of the common model structure. Models 1 and 2 are distinguished by the value of $\boldsymbol{f}_{\boldsymbol{A}}$ (model 1: $\boldsymbol{f}_{\boldsymbol{A}}\boldsymbol{=0}$; model 2: $\boldsymbol{f}_{\boldsymbol{A}}\boldsymbol{=1}$). In model 3 ($\boldsymbol{f}_{\boldsymbol{A}}\boldsymbol{=0}$), the infectivity of asymptomatic cases infections is set to zero, such that PKDL is the main reservoir of infection.


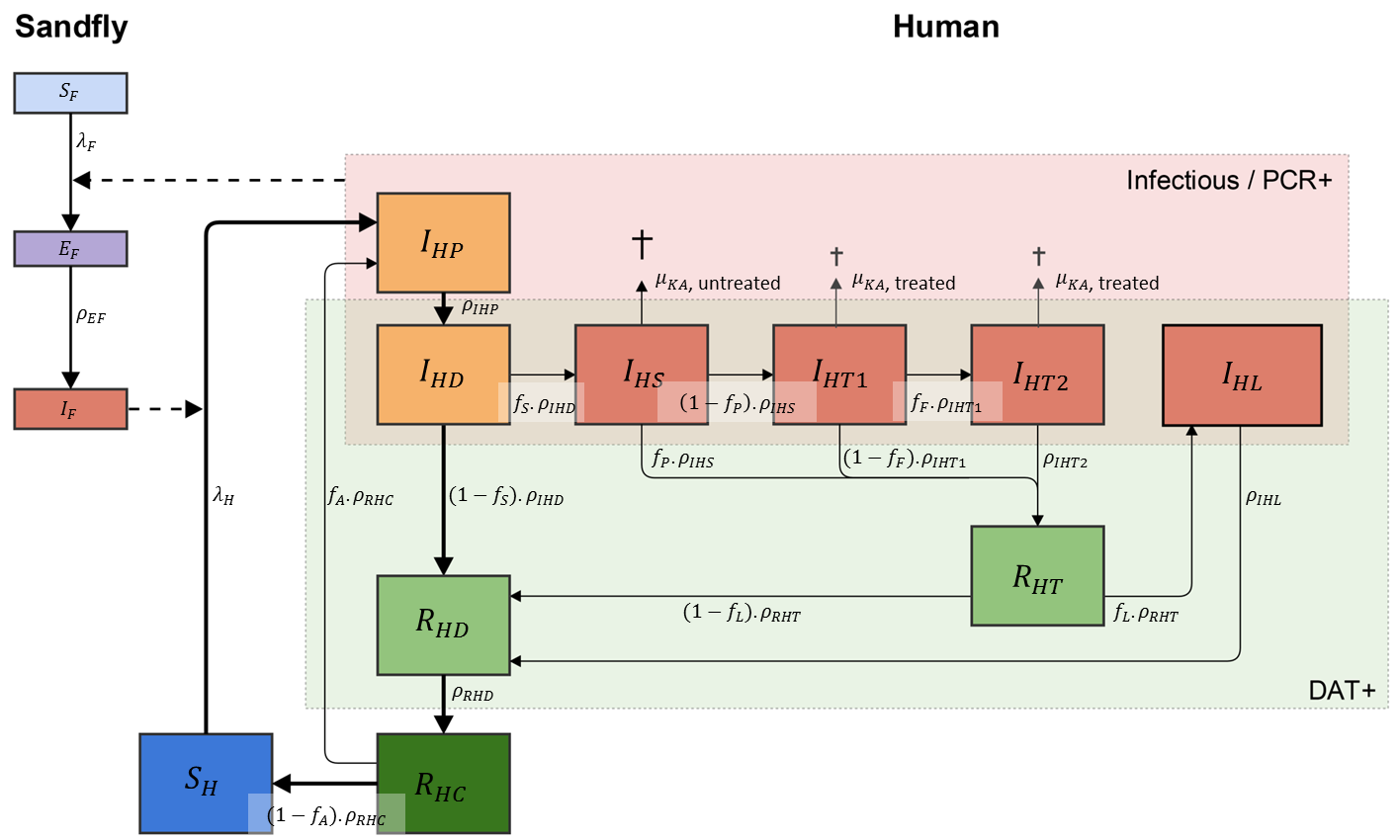


# Data

In the main manuscript, we describe how a subset of model parameters were preset based on literature and published data sources, and how duration of the late recovered stage and age-patterns in exposure were arrived at. The remaining parameters – number of sandflies per human ($N_{F}$), duration of asymptomatic stages of infection ($1/\rho_{IHP}$, $1/\rho_{IHD}$, $1/\rho_{RHD}$), infectivity of human stages of infection towards the sandfly ($p_{IHP}$ and $p_{IHD}$ in models 1 and 2, $p_{IHL}$ in model 3), and the proportion of asymptomatic infections that develop into VL ($f_{S}$) – were estimated based on data from the KalaNet study, a community-based intervention trial in hyperendemic clusters in Bihar, India, and in the Terai plains in Nepal. The KalaNet data constitute cross-sectional information on DAT status (direct agglutination test for presence of antibodies against the parasite) of 21,204 individuals from three time points spanning two years, and information on incidence of VL during the entire two-year study period. For 668 individuals aged 14 and older, PCR testing for detection of parasite DNA was also performed. Further, for a subset of individuals, data from consecutive time points are available, allowing derivation of changes in PCR and DAT status. To quantify our model, we used prevalence of DAT-positivity (titre > 1:800 as the smallest serum dilution ratio that still gives a positive result), PCR-positivity, PCR/DAT positivity, incidence of VL and PCR-positivity (i.e. a change from PCR-negative to positive between two consecutive years), and the prevalence of *L. donovani* in sandflies in Nepal. Table A1-2 provides an overview of the KalaNet data.

Table A1-2. Overview of the KalaNet data, aggregated to the population-level. For incidence figures, raw data represent the number of pairs of measurements over two consecutive years (change form negative to positive/total). For prevalence figures, raw data represent the number of measurements performed during all of the three time points. DAT-positivity is defined as a titre > 1:800.

| Metric | Nepal | |  | India | |  | Total | |
| --- | --- | --- | --- | --- | --- | --- | --- | --- |
|  | Point estimate | Raw data |  | Point estimate | Raw data |  | Point estimate | Raw data |
| Incidence of kala-azar (treated cases per 10,000 capita per year) | 13 | 20/15810 |  | 29 | 78/26594 |  | 23 | 98/42404 |
| Incidence of PCR seroconversion (per 10,000 capita per year) | 669 | 20/309 |  | 1158 | 34/311 |  | 911 | 54/620 |
| Prevalence of PCR-positivity (%) | 10.2 | 67/658 |  | 16.3 | 129/793 |  | 13.5 | 196/1451 |
| Prevalence of DAT-positivity (%) | 9.3 | 1593/17149 |  | 17.6 | 4651/26425 |  | 14.3 | 6244/43574 |
| Prevalence of PCR-positivity and DAT-positivity (%) | 2.0 | 13/657 |  | 4.9 | 39/789 |  | 3.6 | 52/1446 |
| Prevalence of infection in sandflies (%)* | 0.5 | - |  | ** | - |  | - | - |
| * In the analysis, we interpret this quantity as the prevalence of infective flies $I_{F}/N_{F}$.  ** In the main analysis, the prevalence of infection in flies in India was estimated at 0.927%. | | | | | | | | |

# Derivation of equations for the system of ODEs at equilibrium

For convenience, we assume that at endemic equilibrium the size of the human population $N_{H}=1$, such that we can interpret the size of each compartment as the prevalence of that state, and flows as incidence per capita in the whole human population. Further, we account for the human population growth during endemic equilibrium by assuming that (even though $N_{H}=1$) each compartment grows exponentially over time with the same net growth rate equal to the human birth rate per capita $\alpha_{H}$ minus the net mortality rate per capita $\mu_{NH}$. For now, we derive equations for the system of ODEs at equilibrium, ignoring age-dependent survival and age-dependent exposure to infection. We provide an example calculation for a fictional human compartment $\text{X}$ at equilibrium:

$$\frac{dX_{H}}{dt}=\text{inflow}_{XH}-\left( \rho_{XH}+\mu_{H}+\mu_{XH} \right)\cdot X_{H}=\left( \alpha_{H}-\mu_{NH} \right)\cdot X_{H}$$

From this, we derive:

$$\text{inflow}_{XH}-\left( \rho_{XH}+\mu_{H}+\mu_{XH}+\alpha_{H}-\mu_{NH} \right)\cdot X_{H}=0$$

Here, $\text{inflow}_{XH}$ is the incidence of $X_{H}$; $\rho_{XH}$ is the per capita rate at which humans move to other compartments; $\mu_{H}$ is the background mortality rate per capita; $\mu_{XH}$ is excess mortality specifically for compartment $X_{H}$ (if applicable); $\alpha_{H}$ is the per capita birth rate; and $\mu_{NH}$ is the net mortality rate per capita over the whole population $N_{H}$ (i.e. the sum of all background and excess mortality in the population in absolute terms, divided by $N_{H}$). Now we define the size of compartment $X_{H}$ in an equilibrium situation:

$$X_{H}=\frac{\text{inflow}_{XH}}{\rho_{XH}+\mu_{H}+\mu_{XH}+\alpha_{H}-\mu_{NH}}$$

For an analytical solution of $X_{H}$, we simplify the equation by assuming that the level of excess mortality in the entire human populations is negligible compared to overall mortality ($\mu_{NH}=\mu_{H}$, which is reasonable as kala-azar is rare), such that:

$$X_{H}=\frac{\text{inflow}_{XH}}{\rho_{XH}+\mu_{XH}+\alpha_{H}}$$

In the next subsection, we start deriving the equilibrium equations at the flow into the compartment for first-line treatment of kala-azar cases *I_HT_*_1_, for which we know the rate from the data (i.e. the observed incidence of kala-azar). Given assumptions about the average durations of *I_HT_*_1_ and consecutive clinical compartments up till putatively recovered $R_{HT}$, we will derive equations for the prevalence of all clinical compartments. Given these prevalences, we will backtrack to PCR incidence and derive equations for the duration of the early and late stages of asymptomatic infection (PCR+/DAT- and PCR+/DAT+, respectively), and early recovered humans (PCR-/DAT+), given data on prevalence of PCR+/DAT-, PCR+/DAT+, and PCR-/DAT+ cases. Then given an assumption about the average duration of the late recovered state (PCR-/DAT-), we derive the sizes of the compartments for late recovered and susceptible humans. Last, given the prevalence of infected flies and assumptions about the relative infectiveness of symptomatic human stages of infection, we derive the infectiveness of asymptomatic stages of infection (model 1 and 2) or PKDL (model 3). Data are indicated with square brackets, e.g. “$\left[ \text{incidence of kala-azar} \right]$”.

Each of the following subsections will cover the derivation of equations related to a specific compartment or parameter in the situation where the system of ODEs is at equilibrium. Each subsection starts with relevant equations and then continues to describe the derivation of the solution given data and equations for other compartments and/or parameters. In section 5, we present and summarise results of the calculations.

## First-line treatment of symptomatic cases (PCR+/DAT+)

$$\frac{dI_{HT1}}{dt}=\left( 1-f_{P} \right)\cdot\rho_{IHS}\cdot I_{HS}-\left( \rho_{IHT1}+\mu_{H}+\mu_{KA\text{, treated}} \right)\cdot I_{HT1}=\left( \alpha_{H}-\mu_{H} \right)\cdot I_{HT1}$$

We calculate the prevalence of first-line treatment $I_{HT1}$, given the observed incidence of first-line VL treatments, which is represented by $\left[ \text{incidence of kala-azar} \right]$, and assumptions about average duration of first-line treatment $1/\rho_{IHT1}$, human birth rate $\alpha_{H}$, and excess mortality $\mu_{KA\text{, treated}}$:

$$I_{HT1}=\frac{\left( 1-f_{P} \right)\cdot\rho_{IHS}\cdot I_{HS}}{\rho_{IHT1}+\alpha_{H}+\mu_{KA\text{, treated}}}=\frac{\left[ \text{incidence of kala-azar} \right]}{\rho_{IHT1}+\alpha_{H}+\mu_{KA\text{, treated}}}$$

## Untreated symptomatic cases originating from late asymptomatic infection (PCR+/DAT+)

$$\frac{dI_{HT1}}{dt}=\left( 1-f_{P} \right)\cdot\rho_{IHS}\cdot I_{HS}-\left( \rho_{IHT1}+\mu_{H}+\mu_{KA\text{, treated}} \right)\cdot I_{HT1}=\left( \alpha_{H}-\mu_{H} \right)\cdot I_{HT1}$$

We calculate the prevalence of untreated cases $I_{HS}$ by back-calculating from the equation for prevalence of first-line treatments $I_{HT1}$, given assumptions about the average detection delay $1/\rho_{IHS}$ for untreated cases, fraction of VL cases that spontaneously recover before being detected $f_{P}$, human birth rate $\alpha_{H}$, and excess mortality $\mu_{KA\text{, treated}}$:

$$I_{HS}=\frac{I_{HT1}\cdot\left( \rho_{IHT1}+\alpha_{H}+\mu_{KA\text{, treated}} \right)}{\left( 1-f_{P} \right)\cdot\rho_{IHS}}$$

## Second-line treatment of symptomatic cases (PCR+/DAT+)

$$\frac{dI_{HT2}}{dt}=f_{F}\cdot\rho_{IHT1}\cdot I_{HT1}-\left( \rho_{IHT2}+\mu_{H}+\mu_{KA\text{, treated}} \right)\cdot I_{HT2}=\left( \alpha_{H}-\mu_{H} \right)\cdot I_{HT2}$$

We calculate the prevalence of second-line treatments $I_{HT2}$, given the equation for prevalence of first-line treatments $I_{HT1}$ and assumptions about average duration of first-line treatment $1/\rho_{IHT1}$, proportion of failed first-line treatments $f_{F}$, average duration of second-line treatment $1/\rho_{IHT2}$, and human birth rate $\alpha_{H}$ and excess mortality $\mu_{KA\text{, treated}}$:

$$I_{HT2}=\frac{f_{F}\cdot\rho_{IHT1}\cdot I_{HT1}}{\rho_{IHT2}+\alpha_{H}+\mu_{KA\text{, treated}}}$$

## Putatively recovered (PCR-/DAT+)

$$\frac{dR_{HT}}{dt}=f_{P}\cdot\rho_{IHS}\cdot I_{HS}+\left( 1-f_{F} \right)\cdot\rho_{IHT1}\cdot I_{HT1}+\rho_{IHT2}\cdot I_{HT2}-\left( \rho_{RHT}+\mu_{H} \right)\cdot R_{HT}=\left( \alpha_{H}-\mu_{H} \right)\cdot R_{HT}$$

We calculate the prevalence of putatively recovered cases $R_{HT}$, given spontaneously recovered untreated clinical cases $f_{P}\cdot\rho_{IHS}\cdot I_{HS}$ and new clinical cases successfully treated with first and second-line treatment $\left( 1-f_{F} \right)\cdot\rho_{IHT1}\cdot I_{HT1}+\rho_{IHT2}\cdot I_{HT2}$, and assumptions about the average duration of the putatively recovered state $1/\rho_{RHT}$ and human birth rate $\alpha_{H}$:

$$R_{HT}=\frac{f_{P}\cdot\rho_{IHS}\cdot I_{HS}+\left( 1-f_{F} \right)\cdot\rho_{IHT1}\cdot I_{HT1}+\rho_{IHT2}\cdot I_{HT2}}{\rho_{RHT}+\alpha_{H}}$$

## Post kala-azar dermal leishmaniasis (PCR+/DAT+)

$$\frac{dI_{HL}}{dt}=f_{L}\cdot\rho_{RHT}\cdot R_{HT}-\left( \rho_{IHL}+\mu_{H} \right)\cdot I_{HL}=\left( \alpha_{H}-\mu_{H} \right)\cdot I_{HL}$$

We calculate $I_{HL}$, the prevalence of post kala-azar dermal leishmaniasis (PKDL), given the equation for prevalence of putatively recovered cases $R_{HT}$, and the assumption about the average duration of the putatively recovered state $1/\rho_{RHT}$, the proportion $f_{L}$ of putatively recovered cases that develop PKDL, duration of PKDL $1/\rho_{IHL}$, and human birth rate $\alpha_{H}$:

$$I_{HL}=\frac{f_{L}\cdot\rho_{RHT}\cdot R_{HT}}{\rho_{IHL}+\alpha_{H}}$$

## Early asymptomatic (PCR+/DAT-)

$$\frac{dI_{HP}}{dt}=\lambda_{H}\cdot S_{H}+f_{A}\cdot\rho_{RHC}\cdot R_{HC}-\left( \rho_{IHP}+\mu_{H} \right)\cdot I_{HP}=\left( \alpha_{H}-\mu_{H} \right)\cdot I_{HP}$$

We calculate $\rho_{IHP}$, which is 1/duration of early asymptomatic infection $I_{HP}$, given data on PCR incidence (PCR seroconversion) and the prevalence of PCR+/DAT- serostatus, and our assumption about human birth rate $\alpha_{H}$:

$$\rho_{IHP}=\frac{\lambda_{H}\cdot S_{H}+f_{A}\cdot\rho_{RHC}\cdot R_{HC}}{I_{HP}}-\alpha_{H}=\frac{\left[ \text{incidence of PCR+} \right]}{\left[ \text{prevalence of PCR+/DAT-} \right]}-\alpha_{H}$$

## Late asymptomatic (PCR+/DAT+)

$$\frac{dI_{HD}}{dt}=\rho_{IHP}\cdot I_{HP}-\left( \rho_{IHD}+\mu_{H} \right)\cdot I_{HD}=\left( \alpha_{H}-\mu_{H} \right)\cdot I_{HD}$$

We calculate $\rho_{IHD}$, which is 1/duration of late asymptomatic infection $I_{HD}$, given equations for average duration of early asymptomatic infection $1/\rho_{IHP}$ and prevalence of all clinical cases ($I_{HS}+I_{HT1}+I_{HT2}+I_{HL}$, which are al PCR+/DAT+), data on prevalence of PCR+/DAT- and PCR+/DAT+ cases, and our assumption about human birth rate $\alpha_{H}$:

$$\rho_{IHD}=\frac{\rho_{IHP}\cdot I_{HP}}{I_{HD}}-\alpha_{H}=\frac{\rho_{IHP}\cdot\left[ \text{prevalence of PCR+/DAT-} \right]}{\left[ \text{prevalence of PCR+/DAT+ } \right]-\left( I_{HS}+I_{HT1}+I_{HT2}+I_{HL} \right)}-\alpha_{H}$$

## Fraction of late asymptomatic cases that develop clinical disease

$$\frac{dI_{HS}}{dt}=f_{S}\cdot\rho_{IHD}\cdot I_{HD}-\left( \rho_{IHS}+\mu_{H}+\mu_{KA\text{, untreated}} \right)\cdot I_{HS}=\left( \alpha_{H}-\mu_{H} \right)\cdot I_{HS}$$

We calculate the fraction $f_{S}$ of late asymptomatic cases that develop clinical disease, given data on PCR+/DAT+ positive cases; equations for duration of late asymptomatic cases $1/\rho_{IHD}$, prevalence of untreated clinical cases $I_{HS}$, and prevalence of all clinical stages ($I_{HS}+I_{HT1}+I_{HT2}+I_{HL}$); and assumptions about the duration of the untreated clinical state $1/\rho_{IHS}$, human birth rate $\alpha_{H}$, and excess mortality $\mu_{KA\text{, untreated}}$ among untreated kala-azar cases:

$$f_{S}=\text{ }\frac{\left( \rho_{IHS}+\alpha_{H}+\mu_{KA\text{, untreated}} \right)\cdot I_{HS}}{\rho_{IHD}\cdot I_{HD}}$$

$$f_{S}=\frac{\left( \rho_{IHS}+\alpha_{H}+\mu_{KA\text{, untreated}} \right)\cdot I_{HS}}{\rho_{IHD}\cdot\left( \left[ \text{prevalence of PCR+/DAT+} \right]-\left( I_{HS}+I_{HT1}+I_{HT2}+I_{HL} \right) \right)}$$

## Early recovered (PCR-/DAT+)

$$\frac{dR_{HD}}{dt}=\left( 1-f_{S} \right)\cdot\rho_{IHD}\cdot I_{HD}+\left( 1-f_{L} \right)\cdot\rho_{RHT}\cdot R_{HT}+\rho_{IHL}\cdot I_{HL}-\left( \rho_{RHD}+\mu_{H} \right)\cdot R_{HD}$$

|  | $=\left( \alpha_{H}-\mu_{H} \right)\cdot R_{HD}$ |
| --- | --- |

We calculate $\rho_{RHD}$, which is 1/duration of the early recovered state, given data on prevalence of PCR-/DAT+ serostatus; equations for prevalence of putatively recovered cases $R_{HT}$, prevalence of late asymptomatic infection $I_{HD}$, prevalence of PKDL $I_{HL}$, and the fraction $f_{S}$ of late asymptomatic cases that develop clinical disease; and assumptions about the average duration of late asymptomatic infection ${1/\rho}_{IHD}$, average duration of the putatively recovered state $1/\rho_{RHT}$, average duration of PKDL $1/\rho_{IHL}$, the proportion $f_{L}$ of putatively recovered cases that develop PKDL, and human birth rate $\alpha_{H}$:

$$R_{HD}=\left[ \text{prevalence of PCR-/DAT+} \right]-R_{HT}$$

$$\rho_{RHD}=\frac{\left( 1-f_{S} \right)\cdot\rho_{IHD}\cdot I_{HD}+\left( 1-f_{L} \right)\cdot\rho_{RHT}\cdot R_{HT}+\rho_{IHL}\cdot I_{HL}}{R_{HD}}-\alpha_{H}$$

Note that, because we assume that the system is at equilibrium, the flow into the early recovered stage $R_{HD}$ is equal to the flow into early asymptomatic infection $I_{HP}$ (PCR incidence) minus deaths that occur in the stages between $I_{HP}$ and $R_{HD}$ (not further defined here). We can therefore conclude that under the equilibrium assumption, $\rho_{RHD}$ it is entirely determined by the data on PCR incidence and prevalence of PCR-/DAT+ cases (along with assumptions about duration of clinical stages and human birth and mortality rates):

$$\rho_{RHD}=\frac{\left[ \text{incidence of PCR+ } \right]-\left[ \text{deaths among }I_{HP}\text{, }I_{HD}\text{, }I_{HS}\text{, }I_{HT1}\text{, }I_{HT2}\text{, }R_{HT}\text{, and }I_{HL} \right]}{\left[ \text{prevalence of PCR-/DAT+} \right]-R_{HT}}-\alpha_{H}$$

## Late recovered (PCR-/DAT-)

$$\frac{dR_{HC}}{dt}=\rho_{RHD}\cdot R_{HD}-\left( \rho_{RHC}+\mu_{H} \right)\cdot R_{HC}=\left( \alpha_{H}-\mu_{H} \right)\cdot R_{HC}$$

We calculate the prevalence of late recovered cases $R_{HC}$, given the equations for prevalence and duration of the early recovered state $R_{HD}$ and $1/\rho_{RHD}$, and assumptions about duration of the late recovered state $1/\rho_{RHC}$ and human birth rate $\alpha_{H}$:

$$R_{HC}=\frac{\rho_{RHD}\cdot R_{HD}}{\rho_{RHC}+\alpha_{H}}$$

## Susceptible humans (PCR-/DAT-)

$$\frac{dS_{H}}{dt}= N_{H}\cdot\alpha_{H}+\left( 1-f_{A} \right)\cdot\rho_{RHC}\cdot R_{HC}-\left( \lambda_{H}+\mu_{H} \right)\cdot S_{H}=\left( \alpha_{H}-\mu_{H} \right)\cdot S_{H}$$

We calculate the prevalence of susceptible humans $S_{H}$, given our assumptions about the size of the human population $N_{H}=1$ and equations for the prevalence of all other human compartments:

$$S_{H}=1-\left( I_{HP}+I_{HD}+R_{HD}+R_{HC}+I_{HS}+I_{HT1}+I_{IHT2}+R_{HT}+I_{HL} \right)$$

If at this stage, the solution for $S_{H}$ is negative, this indicates that the model and the underlying assumptions do not support observed infection levels (i.e. there are not enough susceptible people to support transmission at the observed level). The assumed average duration of the late recovered stage $1/\rho_{RHC}$ is the most important bottleneck for the size of the susceptible human population (models 1 and 3) or the incidence of new PCR-positive cases (model 2). In other words, for longer durations of $R_{HC}$, the model will not be able to reproduce the data. The value of $1/\rho_{RHC}$ is counterbalanced by human birth rate $\alpha_{H}$; higher values of $\alpha_{H}$ are associated with and higher prevalence of susceptible humans and lower prevalences of non-susceptible human stages.

## Force of infection acting on humans and number of flies per human

Next, we calculate the force of infection $\lambda_{H}$ acting on humans in two ways, given $S_{H}$ and given the differential equations for $dS_{H}/dt$ and $dI_{HP}/dt$, respectively:

$$\lambda_{H}= \frac{\alpha_{H}+\left( 1-f_{A} \right)\cdot\rho_{RHC}\cdot R_{HC}}{S_{H}}-\alpha_{H}$$

$$\lambda_{H}=\frac{\left( \rho_{IHP}+\alpha_{H} \right)\cdot I_{HP}-f_{A}\cdot\rho_{RHC}\cdot R_{HC}}{S_{H}}$$

The values of these two solutions differ slightly because we assumed that the contribution of excess mortality to the overall mortality in the human population is negligible ($\mu_{NH}=\mu_{H}$), while keeping excess mortality $\mu_{XH}$ at a non-zero value in the denominators of the solutions for prevalence of treated and untreated kala-azar. With the more sophisticated assumption of $\mu_{NH}=\mu_{H}+\sum\mu_{XH}\cdot X_{H}$, the solutions to the two equations for $\lambda_{H}$ are exactly the same, but can only be calculated with numerical methods. Alternatively, excess mortality $\mu_{XH}$ could be assumed to be zero (i.e. removing it from the denominators of the equations for prevalence of treated and untreated kala-azar), which again allows for an analytical solution and identical solutions to the two equations for $\lambda_{H}$.

From the equations for $\lambda_{H}$ we can conclude that the higher the birth rate in the population, the higher the force of infection required to reproduce the levels of infection observed in the data (i.e. to counter the diluting effect of population growth on infection levels). Further, higher human birth rates $\alpha_{H}$ allow for longer duration of late recovery $R_{HC}$ (lower values of $\rho_{RHC}$). If both $\rho_{RHC}$ and $\alpha_{H}$ are too low, $S_{H}$ will be too small to sustain transmission levels (PCR incidence) as observed in the data. As $\rho_{RHC}$ approaches its critical minimum value from above (i.e. longer duration of $R_{HC}$), the force of infection $\lambda_{H}$ will approach positive infinity. Likewise, for any given $\rho_{RHC}$ and $\alpha_{H}$, increases in $\lambda_{H}$ will at some point result in saturation of predicted infections levels at equilibrium through depletion of $S_{H}$.

## Number of flies per human

$$\lambda_{H}=\beta\cdot p_{H}\cdot I_{F}$$

We can now calculate the number of flies per human $N_{F}$, given force of infection $\lambda_{H}$ and the observed prevalence of infection in flies, assuming that the observed prevalence of infection in flies is represented by prevalence of infective flies ($I_{F}/N_{F}$) in the model:

$$\lambda_{H}=\beta\cdot p_{H}\cdot N_{F}\cdot\left[ \text{prevalence of infection in flies} \right]$$

$$N_{F}=\frac{\lambda_{H}}{\beta\cdot p_{H}\cdot\left[ \text{prevalence of infection in flies} \right]}$$

## Infectivity of human infected stages towards the sandfly

$$\frac{dS_{F}}{dt}=\mu_{F}\cdot N_{F}\cdot\left( 1-\text{effect}_{\text{IRS}} \right)-\left( \lambda_{F}+\mu_{F} \right)\cdot S_{F}=0$$

$$\frac{dE_{F}}{dt}=\lambda_{F}\cdot S_{F}-\left( \rho_{EF}+\mu_{F} \right)\cdot E_{F}=0$$

$$\frac{dI_{F}}{dt}=\rho_{EF}\cdot E_{F}-\mu_{F}\cdot I_{F}=0$$

$$\lambda_{F}=\beta\cdot\frac{\sum_{X=I_{HP},\text{ }I_{HD},\text{ }I_{HS},\text{ }I_{HT1},\text{ }I_{HT2},\text{ }I_{HL}} X\cdot p_{X}}{N_{H}}$$

Again, note that for model 3 $p_{IHP}=p_{IHD}=0$.

First, we calculate the size of each sandfly compartment at equilibrium (and in absence of vector control), given the total number of sandflies per human $N_{F}$:

$$S_{F}=\frac{\mu_{F}}{\lambda_{F}+\mu_{F}}\cdot N_{F}$$

$$E_{F}=\frac{\lambda_{F}}{\rho_{EF}+\mu_{F}}\cdot S_{F}$$

$$I_{F}=\frac{\rho_{EF}}{\mu_{F}}\cdot E_{F}=\frac{\rho_{EF}}{\mu_{F}}\cdot\frac{\lambda_{F}}{\rho_{EF}+\mu_{F}}\cdot S_{F}=\frac{\rho_{EF}}{\mu_{F}}\cdot\frac{\lambda_{F}}{\rho_{EF}+\mu_{F}}\cdot\frac{\mu_{F}}{\lambda_{F}+\mu_{F}}\cdot N_{F}=\frac{\rho_{EF}}{\rho_{EF}+\mu_{F}}\cdot\frac{\lambda_{F}}{\lambda_{F}+\mu_{F}}\cdot N_{F}$$

Next, we define prevalence of infective flies in the sandfly population as a function of the force of infection $\lambda_{F}$ acting on the sandfly population:

$$\frac{I_{F}}{N_{F}}=\frac{\rho_{EF}}{\rho_{EF}+\mu_{F}}\cdot\frac{\lambda_{F}}{\lambda_{F}+\mu_{F}}=\left( 1+\frac{\mu_{F}}{\rho_{EF}} \right)^{-1}\cdot\left( 1+\frac{\mu_{F}}{\lambda_{F}} \right)^{-1}$$

Now, given that $\lambda_{F}=\beta\cdot\frac{\sum_{X=I_{HP},\text{ }I_{HD},\text{ }I_{HS},\text{ }I_{HT1},\text{ }I_{HT2},\text{ }I_{HL}} X\cdot p_{X}}{N_{H}}$ and the assumption that $N_{H}=1$:

$$\frac{I_{F}}{N_{F}}=\left( 1+\frac{\mu_{F}}{\rho_{EF}} \right)^{-1}\cdot\left( 1+\frac{\mu_{F}}{\beta\cdot\sum_{X=I_{HP},\text{ }I_{HD},\text{ }I_{HS},\text{ }I_{HT1},\text{ }I_{HT2},\text{ }I_{HL}} X\cdot p_{X}} \right)^{-1}$$

From this, we can conclude that in an equilibrium situation, the prevalence of infective flies in the sandfly population is determined by the infectiveness $p_{X}$ of human stages and assumptions about fly mortality $\mu_{F}$ and duration of latent infection in flies $1/\rho_{EF}$. Further, $N_{F}$ itself also determines the prevalence of infective flies through the overall level of transmission and the resulting levels of infections in humans $p_{X}$. In other words, without data on prevalence of infection in sandflies $I_{F}/N_{F}$, the force of infection $\lambda_{H}$ acting on humans (required for observed infection levels in humans) can be achieved by infinitely many combinations of number of flies per human $N_{F}$ and values for infectiveness of different humans states of infection towards flies $p_{X}$.

Given data on $\left[ I_{F}/N_{F} \right]$, the solution for $I_{F}/N_{F}$ above, and the equation for $\lambda_{F}$, we define:

$$\lambda_{F}=\beta\cdot\sum_{X=I_{HP},\text{ }I_{HD},\text{ }I_{HS},\text{ }I_{HT1},\text{ }I_{HT2},\text{ }I_{HL}} X\cdot p_{X}=\frac{\mu_{F}}{\left[ \frac{N_{F}}{I_{F}} \right]\cdot\frac{\rho_{EF}}{\rho_{EF}+\mu_{F}}-1}$$

All terms on the right-most side of the equation are either provided by the data ($\left[ I_{F}/N_{F} \right]$) or based on assumptions, which allows us to calculate $\lambda_{F}$. Now, for model 1 and 2 we can calculate the infectivity of early and late asymptomatically infected human cases $p_{IHP}$ and $p_{IHD}$, given the solutions for the prevalence of human stages of infection $X$ (relevant stages: $I_{HP}$, $I_{HD}$, $I_{HS}$, $I_{HT1}$, $I_{HT2}$, and $I_{HL}$), assumed values for infectivity of untreated and treated kala-azar ($p_{IHS}=1.0$ and $p_{IHT}=0.5$, respectively) and post kala-azar dermal leishmaniasis ($p_{IHL}=0.5$), and the assumption that early asymptomatic infection $I_{HP}$ is half as infective as late asymptomatic infection $I_{HD}$:

$$p_{IHD}=\frac{\frac{\lambda_{F}}{\beta}-\sum_{X=I_{HS},\text{ }I_{HT1},\text{ }I_{HT2},\text{ }I_{HL}} X\cdot p_{X}}{I_{HD}+\frac{I_{HP}}{2}}=\frac{\frac{\lambda_{F}}{\beta}-\left( 1.0\cdot I_{HS}+0.5\cdot I_{HT1}+0.5\cdot I_{HT2}+0.5\cdot I_{HL} \right)}{I_{HD}+\frac{I_{HP}}{2}}$$

$$p_{IHP}=\frac{p_{IHD}}{2}$$

Similarly, for model 3 we can calculate $p_{IHL}$, assuming that $p_{IHP}=p_{IHD}=0$:

$$p_{IHL}=\frac{\frac{\lambda_{F}}{\beta}-\sum_{X=\text{ }I_{HS},\text{ }I_{HT1},\text{ }I_{HT2}} X\cdot p_{X}}{I_{HL}}=\frac{\frac{\lambda_{F}}{\beta}-\left( 1.0\cdot I_{HS}+0.5\cdot I_{HT1}+0.5\cdot I_{HT2} \right)}{I_{HL}}$$

# R calculations for equilibrium state of system of ODEs

Here, we calculate the parameters and compartment sizes of the system of ODEs for transmission of visceral leishmaniasis on the Indian sub-continent in equilibrium (see section 4 for the derivation of formulae), using different pre-set values for the average duration of the late recovered stage (*R_HC_*). Solutions are based on KalaNet data from India and Nepal, aggregated over countries, villages, ages, sexes, and years (Table A1-2).

To allow for tractable solutions to the equations described in section 4, we assume that excess mortality among treated and untreated cases of kala-azar is zero, which only has a small impact on parameter estimates, as cases of kala-azar only drive about 10% of the overall transmission (see main analysis) and we fix the duration of the different stages of kala-azar a priori (i.e. we do not fit to data on prevalence of clinical stages, but data on PCR/DAT-positive cases in general, of which only a small fraction are clinical cases). In the main manuscript, we describe the results of a more comprehensive numerical solution of an age-structured system of ODEs based on a maximum likelihood analysis of the KalaNet data by country (Table A1-2), and more detailed assumptions about human demography and excess mortality among cases of kala-azar.

Table A1-3 presents the analytical solutions of model 1, 2, and 3 in equilibrium for parameters related to duration and infectivity of the human stages of infection, and the fraction of asymptomatic infections that progress to kala-azar. Parameter estimates were identical for the three models, except for duration of the late asymptomatic infection $I_{HD}$ in model 3, which is lower than in model 1 and 2 due to a longer assumed duration of post kala-azar dermal leishmaniasis ($I_{HL}$, which we also consider to be PCR and DAT-positive). Figure A1-4 illustrates the solution for $N_{F}$ as a function of the pre-set average duration of the late recovered stage $R_{HC}$ for each of the models. The critical duration of $R_{HC}$ beyond which the model can no longer support the data lies somewhere between 11 and 12 years (assuming that the population grows by 25% in 20 years and that the average human life expectancy is 40 years, which approximates our more detailed assumptions about human mortality and a per capita birth rate in the main analysis). If we solve the model only for the (more highly endemic) Indian villages, the critical duration of $R_{HC}$ is lower: between 7 to 8 years.

In Box A1-1, we provide *R*-code to calculate the equilibrium conditions of the system of ODEs, given the data described in Table A1-2. For simplicity, we further assume that the prevalence of infection in flies as observed in Nepal (0.5%) applies to the entire KalaNet study area (an assumption we relax in the main analysis).

Table A1-3. Analytical solutions to equations for parameters of model 1, 2, and 3 at equilibrium, based on the KalaNet data aggregated over Nepal and India. The prevalence of infective flies was assumed to be 0.713%, which is the average of the observed prevalence of infected flies of 0.5% in Nepal, and the prevalence of infective flies in India as estimated in the main analysis. All parameter estimates are independent of the pre-set value for the average duration of the late recovered stage $\boldsymbol{R}_{\boldsymbol{HC}}$, except for parameter $\boldsymbol{N}_{\boldsymbol{F}}$ (not included here, see Figure A1-4).

| Parameter | | State | Model 1 | Model 2 | Model 3 |
| --- | --- | --- | --- | --- | --- |
| Duration (years) | $I_{HL}$ | | 5* | 5* | 15* |
|  | $I_{HP}$ | | 1.118 | 1.118 | 1.118 |
|  | $I_{HD}$ | | 0.400 | 0.400 | 0.391 |
|  | $R_{HD}$ | | 1.216 | 1.216 | 1.216 |
| Infectivity | $I_{HP}$ | | 0.0133 | 0.0133 | 0* |
|  | $I_{HD}$ | | 0.0265 | 0.0265 | 0* |
|  | $I_{HL}$ | | 0.5* | 0.5* | 2.0160 |
|  | $I_{HS}$ | | 1.0* | 1.0* | 1.0* |
|  | $I_{HT1}$ and $I_{HT2}$ | | 0.5* | 0.5* | 0.5* |
| Proportion of late asymptomatic infections that develop into kala-azar ($f_{S}$) | | | 2.7% | 2.7% | 2.7% |
| * Pre-set values. | | | | | |

Figure A1-5. Association between number of flies $\boldsymbol{N}_{\boldsymbol{F}}$ and the pre-set average duration of the late recovered stage $\boldsymbol{R}_{\boldsymbol{HC}}$ in model 1 and 3 (red line) and model 2 (green line). The results are based on the entire KalaNet dataset, aggregated over geography, year, age, and sex. If only the Indian data are used (where VL incidence was higher), the asymptote lies between 7 and 8 years duration of the late recovered stage.

Box A1-1. *R*-code to calculate the equilibrium conditions of the system of ODEs, given the KalaNet data and assumptions about duration of clinical stages and late recovered stage $\boldsymbol{R}_{\boldsymbol{HC}}$. To allow for tractable solutions to all equations, we assume that excess mortality among treated and untreated cases of kala-azar is zero, which will only have a small impact on parameter estimates, as cases of kala-azar only drive as little as 10% of the overall transmission and we fix the duration of the different stages of kala-azar a priori. For infectivity of human stages of infection, it suffices to define either $\boldsymbol{p}_{\boldsymbol{IHP}}$ and $\boldsymbol{p}_{\boldsymbol{IHD}}$ or $\boldsymbol{p}_{\boldsymbol{IHL}}$, depending on what model is considered (model 1, 2, or 3).

| # Predefine data.  pcr.inc <- -log(1-54/620) # PCR seroconversion rate in the population.  pcr.prev <- 196/1451 # PCR+ prevalence in the population.  pcr.dat.prev <- 52/1446 # PCR+/DAT+ prevalence in the population.  dat.prev <- 6244/43574 # DAT+ prevalence in the population.  ka.inc <- -log(1-98/42404) # Incidence of first-line treatment of kala-  # azar (KA) in the population.  if.prev <- 713e-5 # Prevalence of infection in sandflies  # (infective flies only); average of Indian  # and Nepali data from main analysis.  # Predefine constants.  model <- "Model 1" # "Model 1", "Model 2", or "Model 3".  mu.H <- 1/40 # 1/Human life expectancy at birth  birth.H <- mu.H*1.25^(1/20) # Per capita human birth rate, assuming 25%  # growth in 20 years.  mu.K <- 0 # Excess mortality rate among untreated KA  mu.KT <- 0 # Excess mortality rate among treated KA  rho.IHS <- 365/30 # 1/Duration of untreated KA  rho.IHT1 <- 365/30 # 1/Duration of first-line treated KA  rho.IHT2 <- 365/30 # 1/Duration of second-line treated KA  rho.RHT <- 12/21 # 1/Duration of putatively recovered state  rho.IHL <- 1/5 # 1/Duration of PKDL  rho.RHC <- 1/(1:12) # 1/Duration of late recovered state    f.P <- 1/34 # Fraction of spontaneously recovered KA  f.F <- 5e-2 # Fraction of failed first-line treatment  f.L <- 5e-2 # Fraction of KA cases that develop PKDL  f.A <- ifelse(model == "Model 2", 1, 0)  # Fraction of infections that reactivate    Beta <- 365/4 # Sandfly biting rate  mu.F <- 365/14 # Sandfly mortality rate  rho.EF <- 365/5 # 1/Duration of latent infection in sandflies  p.H <- 1 # Transmission probability of flies to humans    p.IHP <- ifelse(model == "Model 3", 0, 0.0125)  # Relative infectivity of early asymptomatics  p.IHD <- ifelse(model == "Model 3", 0, 0.025)  # Relative infectivity of late asymptomatics  p.IHL <- ifelse(model == "Model 3", 1.0, 0.5)  # Relative infectivity of PKDL  # One or more of the above will be re-estimated  # from data, depending on the model.  p.IHS <- 1 # Relative infectivity of untreated KA  p.IHT1 <- 1 # Relative infectivity of first-line treated KA  p.IHT2 <- 1 # Relative infectivity of 2nd-line treated KA    # Set prevalence of clinical stages, based on observed kala-azar incidence  # and assumed durations and mortality rates.  IHT1 <- ka.inc / c(rho.IHT1 + birth.H + mu.KT)  IHS <- (rho.IHT1 + birth.H + mu.KT) * IHT1 / ((1-f.P) * rho.IHS)  IHT2 <- (f.F * rho.IHT1 * IHT1) / (rho.IHT2 + birth.H + mu.KT)  RHT <- (f.P * rho.IHS * IHS + (1-f.F) * rho.IHT1 * IHT1 +  rho.IHT2 * IHT2) / (rho.RHT + birth.H)  IHL <- f.L * rho.RHT * RHT / (rho.IHL + birth.H)  # Calculate durations of pre-clinical stages and fraction f.S.  IHP <- pcr.prev - pcr.dat.prev  rho.IHP <- pcr.inc / IHP - birth.H  IHD <- pcr.dat.prev - (IHT1 + IHT2 + IHS + IHL)  rho.IHD <- (rho.IHP * IHP) / IHD - birth.H  f.S <- (rho.IHS + birth.H + mu.K) * IHS / (rho.IHD * IHD)  # Calculate durations and prevalence of post-clinical stages.  RHD <- dat.prev - pcr.dat.prev - RHT  rho.RHD <- ((1-f.S) * rho.IHD * IHD + (1-f.L) * rho.RHT * RHT +  rho.IHL * IHL) / RHD - birth.H  RHC <- rho.RHD * RHD / (rho.RHC + birth.H)    # Calculate susceptible population and force of infection (two ways).  SH <- 1 - (IHP + IHD + RHD + RHC + IHS + IHT1 + IHT2 + RHT + IHL)  lambda1.H <- (birth.H + (1-f.A) * rho.RHC * RHC) / SH - birth.H  lambda2.H <- (pcr.inc - f.A * rho.RHC * RHC) / SH  # Error due to computational precision and possibly, ignoring excess  # mortality in the calculation of net population growth rate (if excess  # mortality is not set to zero)  error.lambda.H <- lambda1.H - lambda2.H  # Calculate number of flies per human, given prevalence of infection in  # flies (data) and required force of infection acting on humans  NF <- lambda1.H / (Beta * p.H * if.prev)  # Calculate force of infection towards flies required to achieve target  # prevalence of infection in sandflies  lambda.F <- mu.F / (1/(if.prev) * rho.EF / (rho.EF + mu.F) - 1)    # Calculate infectivity of reservoir of infection  if(model == "Model 3") {  p.IHL <- (lambda.F/Beta - (p.IHP * IHP + p.IHD * IHD +  p.IHS * IHS + p.IHT1 * IHT1 + p.IHT2 * IHT2)) / IHL  } else {  p.IHD <- (lambda.F/Beta - (p.IHS * IHS + p.IHT1 * IHT1 +  p.IHT2 * IHT2 + p.IHL * IHL)) / (IHD + IHP/2)  p.IHP <- p.IHD / 2  }    # Return main assumptions and results  list(  model = model,  birth.H = birth.H,  mu.K = mu.K,  mu.KT = mu.KT,  f.A = f.A,  duration.RHC = 1 / rho.RHC,  duration.IHL = 1 / rho.IHL,  p.IHP = p.IHP,  p.IHD = p.IHD,  p.IHL = p.IHL,  NF = NF,  rho.IHP = rho.IHP,  rho.IHD = rho.IHD,  rho.RHD = rho.RHD,  f.S = f.S,  KA = IHS + IHT1 + IHT2,  IHL = IHL,  lambda.F = lambda.F,  lambda.IHP = Beta * p.IHP * IHP / lambda.F,  lambda.IHD = Beta * p.IHD * IHD / lambda.F,  lambda.KA = Beta * (p.IHS*IHS + p.IHT1*IHT1 + p.IHT2*IHT2)/lambda.F,  lambda.IHL = Beta * p.IHL * IHL / lambda.F,  error.lambda.H = error.lambda.H) |
| --- |

## Scenario analysis for reservoir of infection in symptomatic cases only

As noted in section 4.14, without data on prevalence of infection in sandflies $I_{F}/N_{F}$, the force of infection $\lambda_{H}$ acting on humans can be achieved by infinitely many combinations of number of flies per human $N_{F}$ and values for infectivity of humans towards flies $p_{X}$. For the moment, let us ignore the data on prevalence of infection in sandflies. We can then explore the scenario in which only symptomatic human cases of infection are infective towards the sandfly, assuming (for now) that $p_{IHS}=1.0$ and $p_{IHT1}=p_{IHT2}=p_{IHL}=0.5$, and that asymptomatic cases of infection do not contribute to transmission ($p_{IHP}=p_{IHD}=0$). We start assuming that we have calculated the prevalence of all human states of infection and the force of infection acting on humans $\lambda_{H}$ (required to generate the observed PCR incidence), and continue to derive the number of infected flies per human $I_{F}$ and total number of flies per human $N_{F}$:

$$I_{F}=\frac{\lambda_{H}}{\beta\cdot p_{H}}$$

$$E_{F}=I_{F}\cdot\frac{\mu_{F}}{\rho_{EF}}$$

$$\lambda_{F}=\beta\cdot\sum_{X=\text{ }I_{HS},\text{ }I_{HT1},\text{ }I_{HT2},\text{ }I_{HL}} X\cdot p_{X}$$

$$S_{F}=E_{F}\cdot\frac{\left( \rho_{EF}+\mu_{F} \right)}{\lambda_{F}}$$

$$N_{F}=S_{F}\cdot\frac{\left( \lambda_{F}+u_{F} \right)}{\mu_{F}}=I_{F}+E_{F}+S_{F}$$

This allows us to calculate the (counterfactual) prevalence of infection in sandflies $I_{F}/N_{F}$ under the assumption that asymptomatic human cases of infection do not contribute to infection. When using only the data from Nepal (Table A1-2), and assuming that the duration of $R_{HC}$ is two years, we arrive at an estimated prevalence of 0.077%, which is 6.5 times lower than the observed prevalence of infection in sandflies. We could arrive at a slightly higher estimate if we assume that the infectiveness of treated VL cases and PKDL is also 1.0 (like untreated VL cases), and that both infected (exposed $E_{F}$) and infective flies ($I_{F}$) contribute towards the prevalence of infection in sandflies; however, this only results in an estimated prevalence of infected flies of 0.017%, which is still 2.9 times lower than the observed values. This indicates that under the assumptions of an endemic equilibrium and homogeneous mixing of human and sandfly population, there has to be an additional reservoir of infection in humans to explain the “relatively” high prevalence of infection observed in sandflies.

## Scenario analysis for imperfect DAT testing

We explored the sensitivity of predicted trends in VL incidence (first-line treatments) during IRS for assumptions about sensitivity (70%, 80%, 90%, 95%, or 100%) and specificity of DAT testing (90%, 95%, or 100%) by recalculating the analytical solutions of a simplified set of models (described in the first part of this section). Figure A1-6 illustrates how predicted trends in VL incidence during IRS (assuming 63% reduction in sandfly density, as in the main analysis) are invariant under alternative assumptions about sensitivity and specificity of DAT testing. The main explanation for this is that under imperfect sensitivity and specificity, misclassified cases are simply attributed to a different compartment in the cycle of early asymptomatic infection, late asymptomatic infection, and early recovered. As a result, the total duration of this part of the transmission cycle does do not change and transmission dynamics remain largely the same (apart from a different proportion $f_{S}$ of asymptomatic infections that eventually develop symptoms.

## Scenario analysis for imperfect PCR testing

As for imperfect DAT testing, we explored the sensitivity of predicted trends in VL incidence (first-line treatments) during IRS for assumptions about sensitivity (70%, 80%, 90%, 95%, or 100%) and specificity of PCR testing (98%, 99%, or 100%) by recalculating the analytical solutions of a simplified set of models (described in the first part of this section). Figure A1-7 illustrates how predicted trends in VL incidence during IRS (assuming 63% reduction in sandfly density, as in the main analysis) vary little under alternative assumptions about sensitivity and specificity of PCR testing. The main explanation for this is that sensitivity and specificity of PCR testing affect observed PCR prevalence and incidence in the same manner (lower sensitivity means that true prevalence and incidence are higher than observed; lower specificity means that true prevalence and incidence are lower than observed). Only because this effect is stronger for observed PCR incidence than prevalence (the first involves two measurements), the duration of the early asymptomatic stage $I_{HP}$ varies (and thus transmission dynamics) under alternative assumption about sensitivity and specificity of PCR testing (but only so for values far from 100%). Further, imperfect specificity and sensitivity appear to counter-balance one another.

Figure A1-6. Sensitivity of predicted trends in VL incidence during IRS for assumed sensitivity and specificity of DAT testing. The horizontal dashed line represents the target VL incidence of <1 per 10,000 capita. The pre-control situation was quantified by fitting model parameters to the KalaNet data (aggregated over India and Nepal, as described in section 5), given varying assumptions about DAT sensitivity (70%, 80%, 90%, 95%, or 100%, represented by different lines within panels, which often overlay each other) and specificity (90%, 95%, or 100%, represented by different rows of panels). For the predicted trends in VL incidence, IRS was assumed to reduce the sandfly density by 63% (as in the main analysis). The underlying system of ordinary differential equations represents a simplified set of models without age-structure (i.e. exponential human survival and equal exposure to sandflies for all ages).

Figure A1-7. Sensitivity of predicted trends in VL incidence during IRS for assumed sensitivity and specificity of PCR testing. The horizontal dashed line represents the target VL incidence of <1 per 10,000 capita. The pre-control situation was quantified by fitting model parameters to the KalaNet data (aggregated over India and Nepal, as described in section 5), given varying assumptions about PCR sensitivity (70%, 80%, 90%, 95%, or 100%, represented by different lines within panels, which often overlay each other) and specificity (98%, 99%, or 100%, represented by different rows of panels). For the predicted trends in VL incidence, IRS was assumed to reduce the sandfly density by 63% (as in the main analysis). The underlying system of ordinary differential equations represents a simplified set of models without age-structure (i.e. exponential human survival and equal exposure to sandflies for all ages).

# References

1. Stauch A, Sarkar RR, Picado A, Ostyn B, Sundar S, Rijal S, Boelaert M, Dujardin J-C, Duerr H-P: **Visceral Leishmaniasis in the Indian Subcontinent: Modelling Epidemiology and Control**. *PLoS Negl Trop Dis* 2011, **5**:e1405.
