# Supplementary material for: Feasibility of eliminating visceral leishmaniasis from the Indian subcontinent: explorations with a set of deterministic age-structured transmission models
Source: Parasit Vectors. 2016 Jan 19;9:24. doi: 10.1186/s13071-016-1292-0 (PMC4717541; doi:10.1186/s13071-016-1292-0)
Supplement: Additional file 2: — Supplementary figure illustrating the fit of all model sub-variants to all data types (extended version of Fig. 2 in the main manuscript). (PDF 95 kb) [file 13071_2016_1292_MOESM2_ESM.pdf]

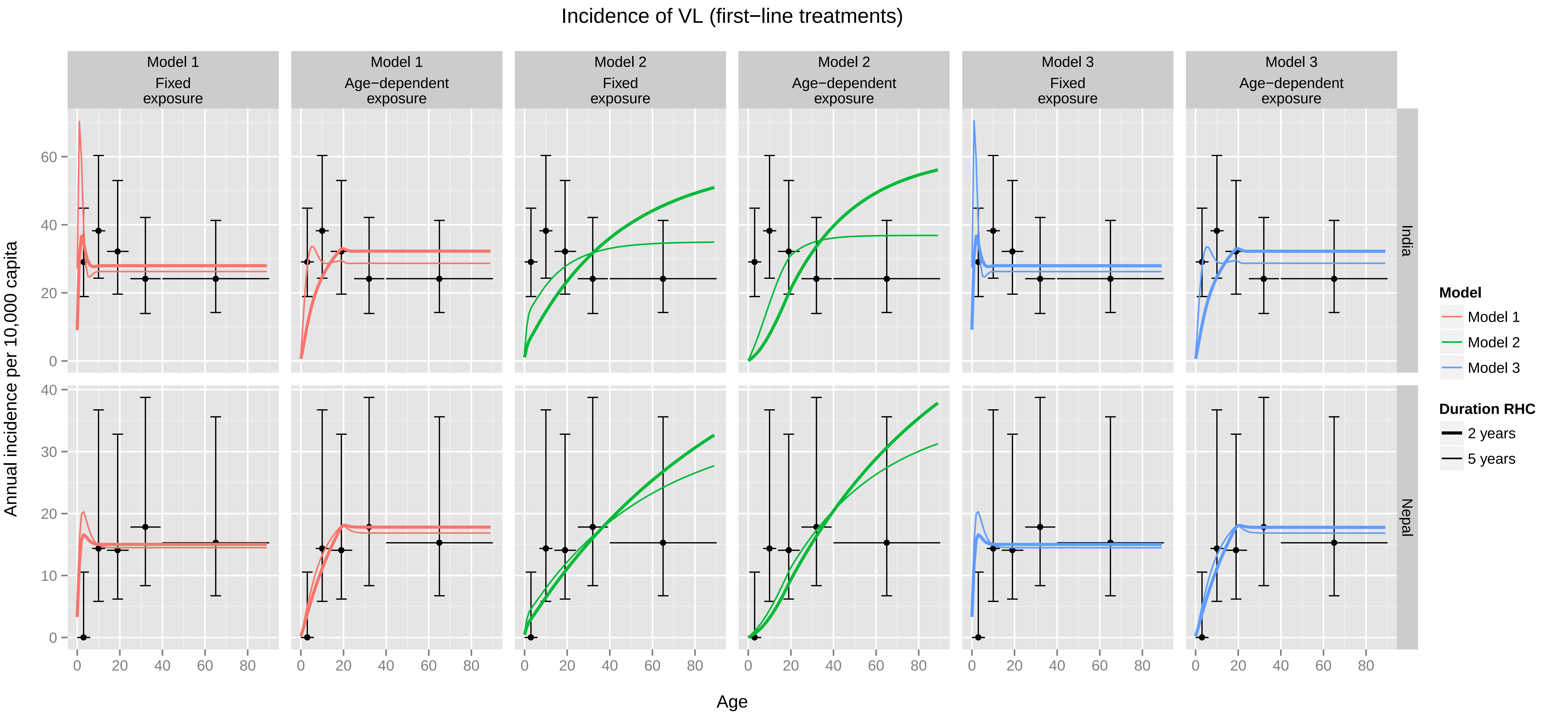

Incidence of VL (first−line treatments)
Model 1
Fixed exposure
Model 1
Age−dependent exposure
Model 2
Fixed exposure
Model 2
Age−dependent exposure
Model 3
Fixed exposure
Model 3
Age−dependent exposure
India
Nepal
Annual incidence per 10,000 capita
Age
0
20
40
60
80
Model
Model 1
Model 2
Model 3
Duration RHC
2 years
5 years

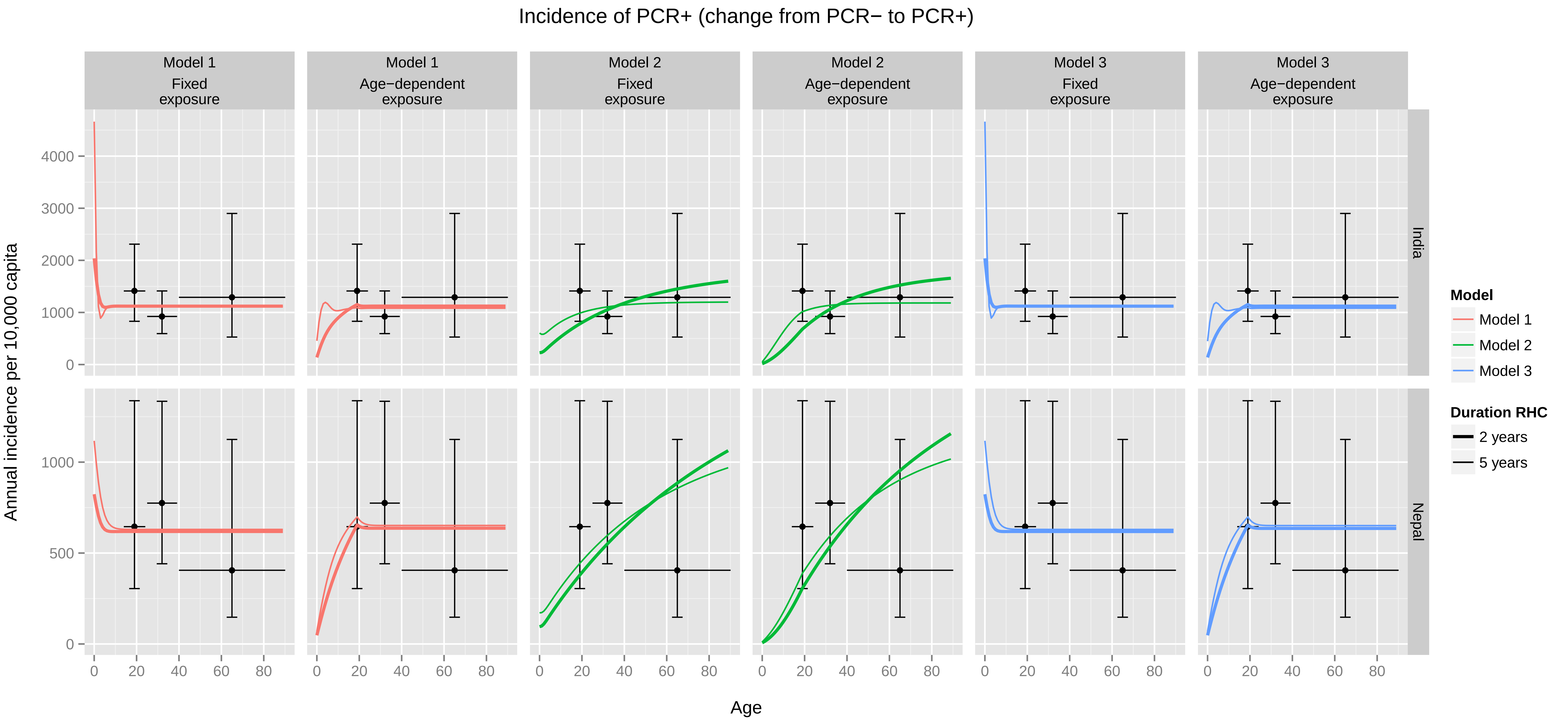

Incidence of PCR+ (change from PCR− to PCR+)
Model 1 Fixed exposure
Model 1 Age−dependent exposure
Model 2 Fixed exposure
Model 2 Age−dependent exposure
Model 3 Fixed exposure
Model 3 Age−dependent exposure
India
Nepal
Annual incidence per 10,000 capita
Age
0
1000
2000
3000
4000
0
500
1000
0
20
40
60
80
Model
Model 1
Model 2
Model 3
Duration RHC
2 years
5 years

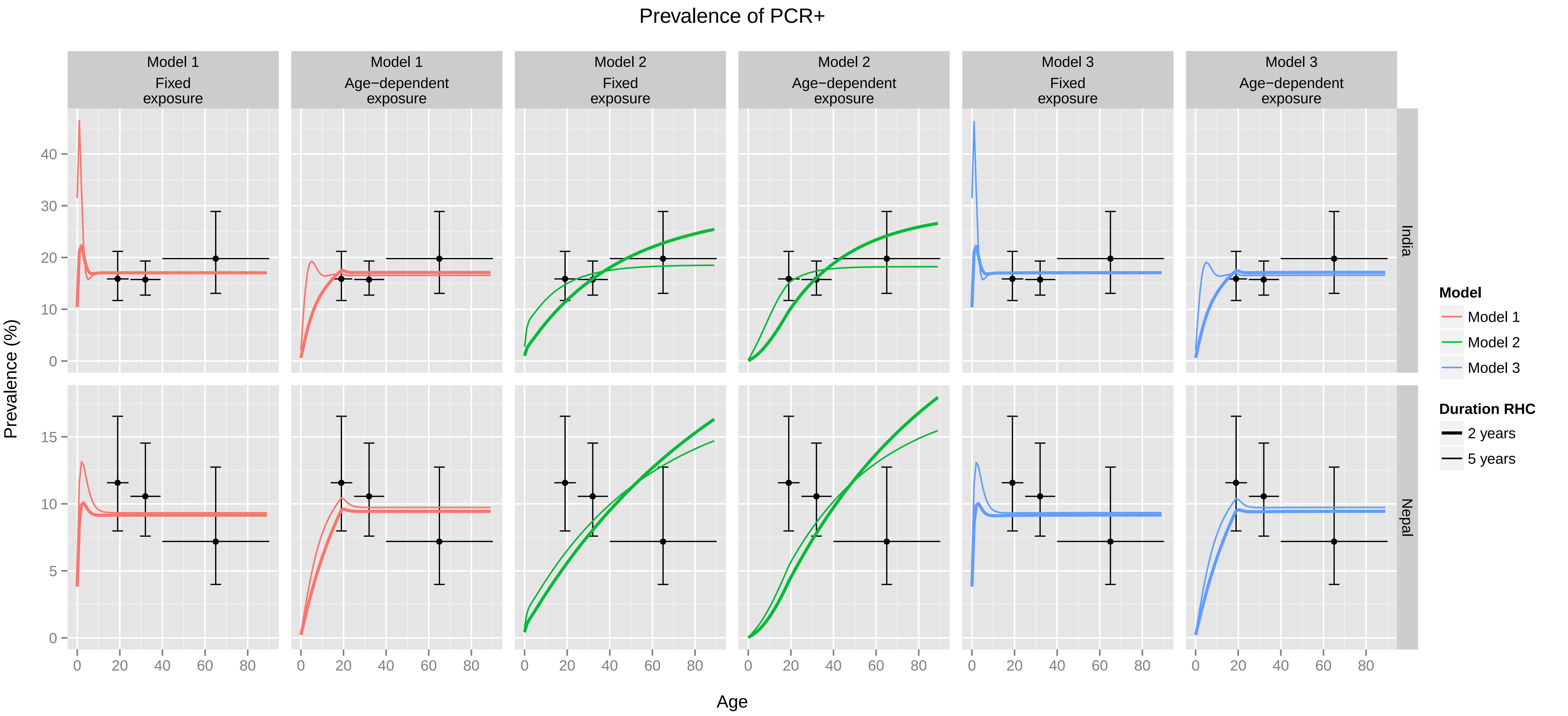

Prevalence of PCR+
Model 1 Fixed exposure
Model 1 Age−dependent exposure
Model 2 Fixed exposure
Model 2 Age−dependent exposure
Model 3 Fixed exposure
Model 3 Age−dependent exposure
India
Nepal
Prevalence (%)
Age
0
10
20
30
40
5
15
60
80
Model
Model 1
Model 2
Model 3
Duration RHC
2 years
5 years

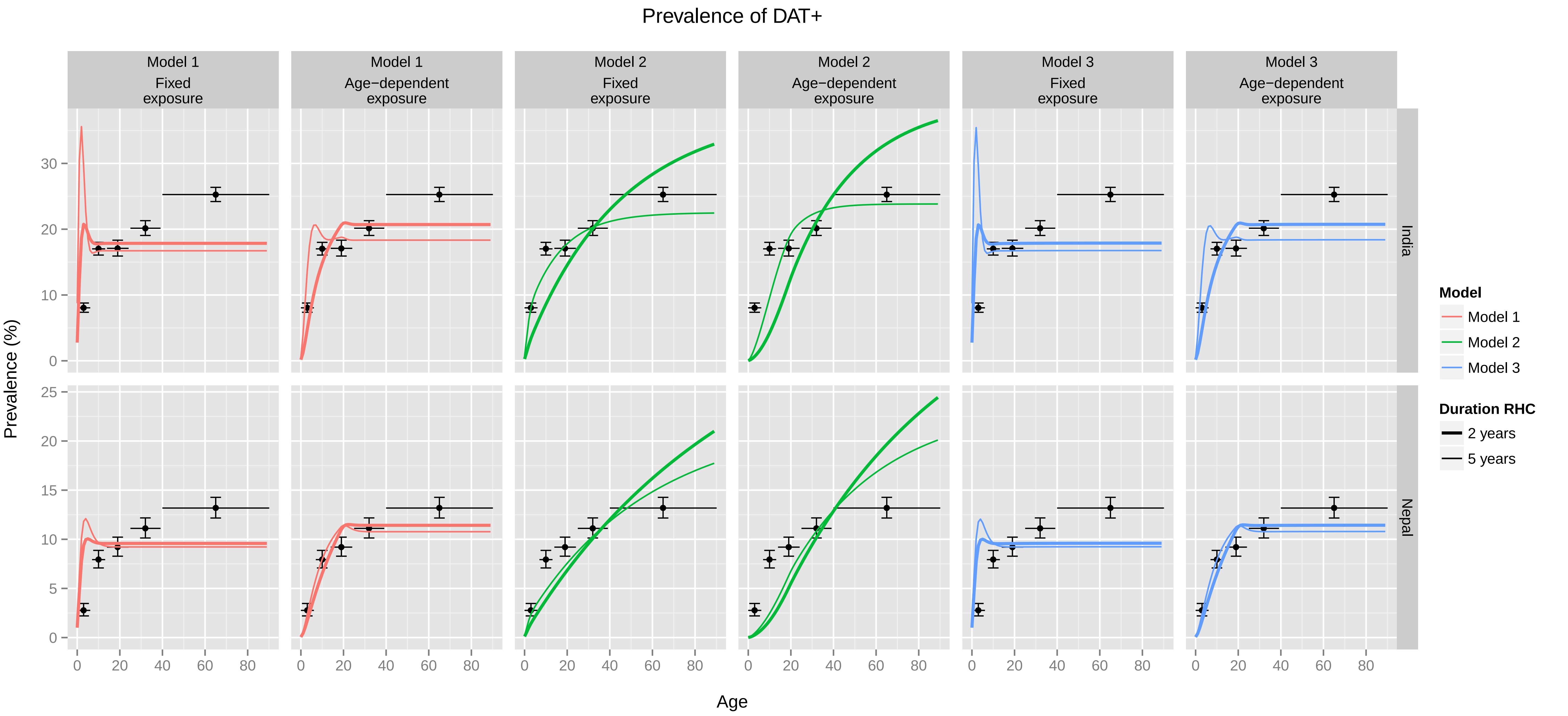

Prevalence of DAT+
Model 1 Fixed exposure
Model 1 Age−dependent exposure
Model 2 Fixed exposure
Model 2 Age−dependent exposure
Model 3 Fixed exposure
Model 3 Age−dependent exposure
India
Nepal
Prevalence (%)
Age
Model
Model 1
Model 2
Model 3
Duration RHC
2 years
5 years

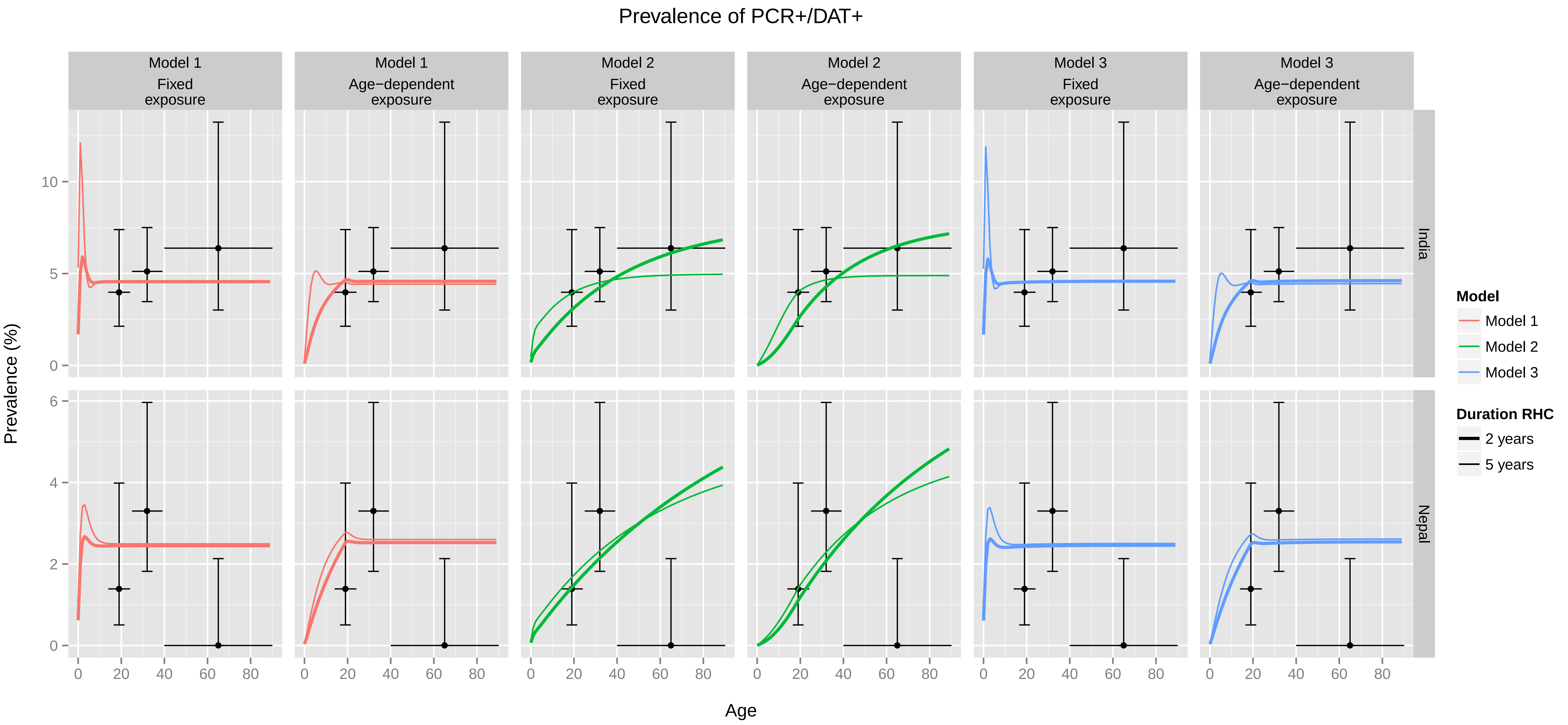
Prevalence of PCR+/DAT+
Model 1
Fixed exposure
Model 1
Age−dependent exposure
Model 2
Fixed exposure
Model 2
Age−dependent exposure
Model 3
Fixed exposure
Model 3
Age−dependent exposure
India
Nepal
Prevalence (%)
Age
0
5
10
0
2
4
6
0
20
40
60
80
Model
Model 1
Model 2
Model 3
Duration RHC
2 years
5 years
