# Supplementary material for: Feasibility of eliminating visceral leishmaniasis from the Indian subcontinent: explorations with a set of deterministic age-structured transmission models
Source: Parasit Vectors. 2016 Jan 19;9:24. doi: 10.1186/s13071-016-1292-0 (PMC4717541; doi:10.1186/s13071-016-1292-0)
Supplement: Additional file 3: — Supplementary figure illustrating the long-term and short-term impact of optimal and sub-optimal IRS in low, medium, and highly endemic areas for all model variants (extended version of Fig. 3 in the main manuscript), as well as transmission dynamics when infection is introduced in a susceptible human population (outbreak). (PDF 7486 kb) [file 13071_2016_1292_MOESM3_ESM.pdf]

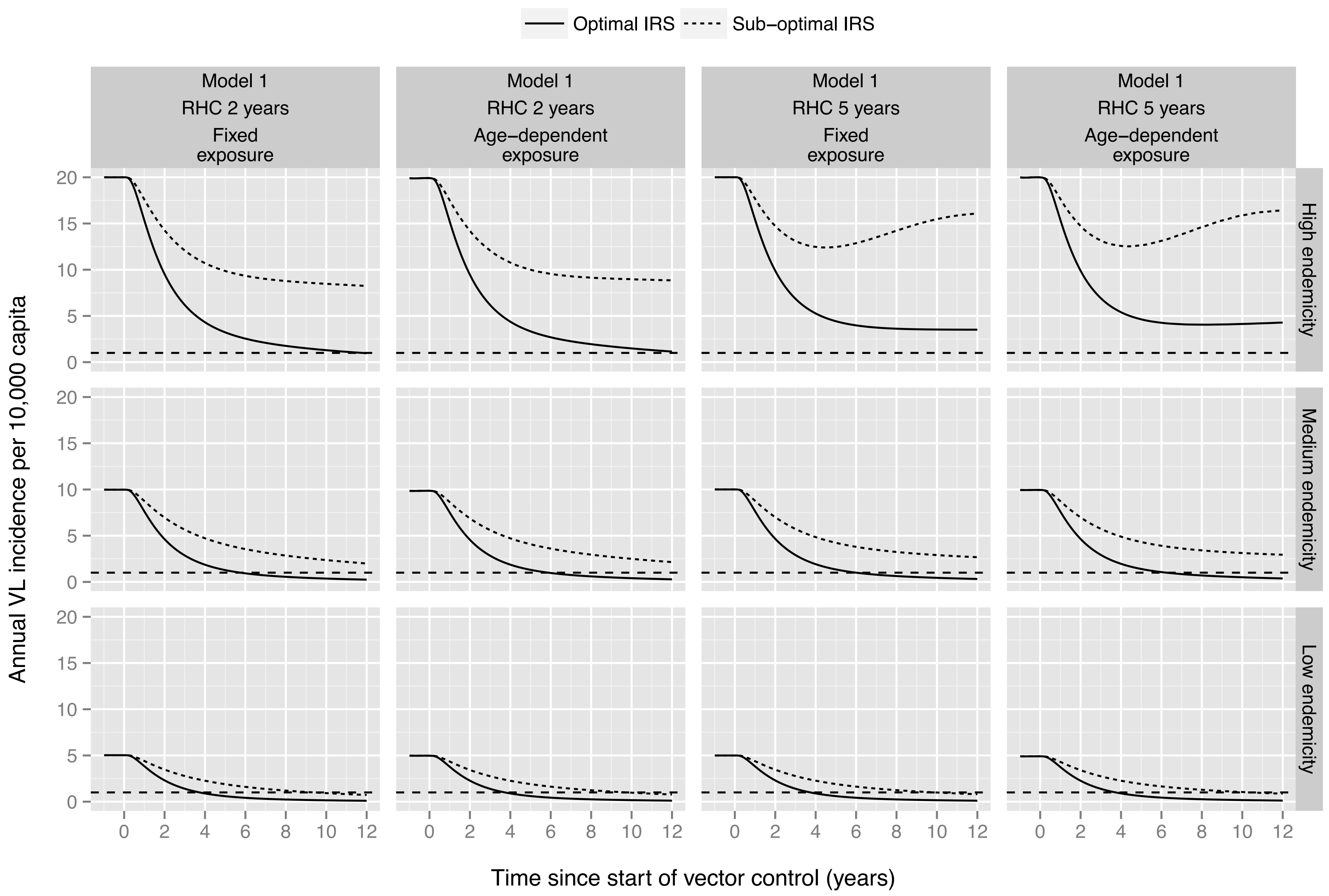

Optimal IRS
Sub−optimal IRS
Model 1
RHC 2 years
Fixed exposure
Model 1
RHC 2 years
Age−dependent exposure
Model 1
RHC 5 years
Fixed exposure
Model 1
RHC 5 years
Age−dependent exposure
High endemicity
Medium endemicity
Low endemicity
Annual VL incidence per 10,000 capita
Time since start of vector control (years)

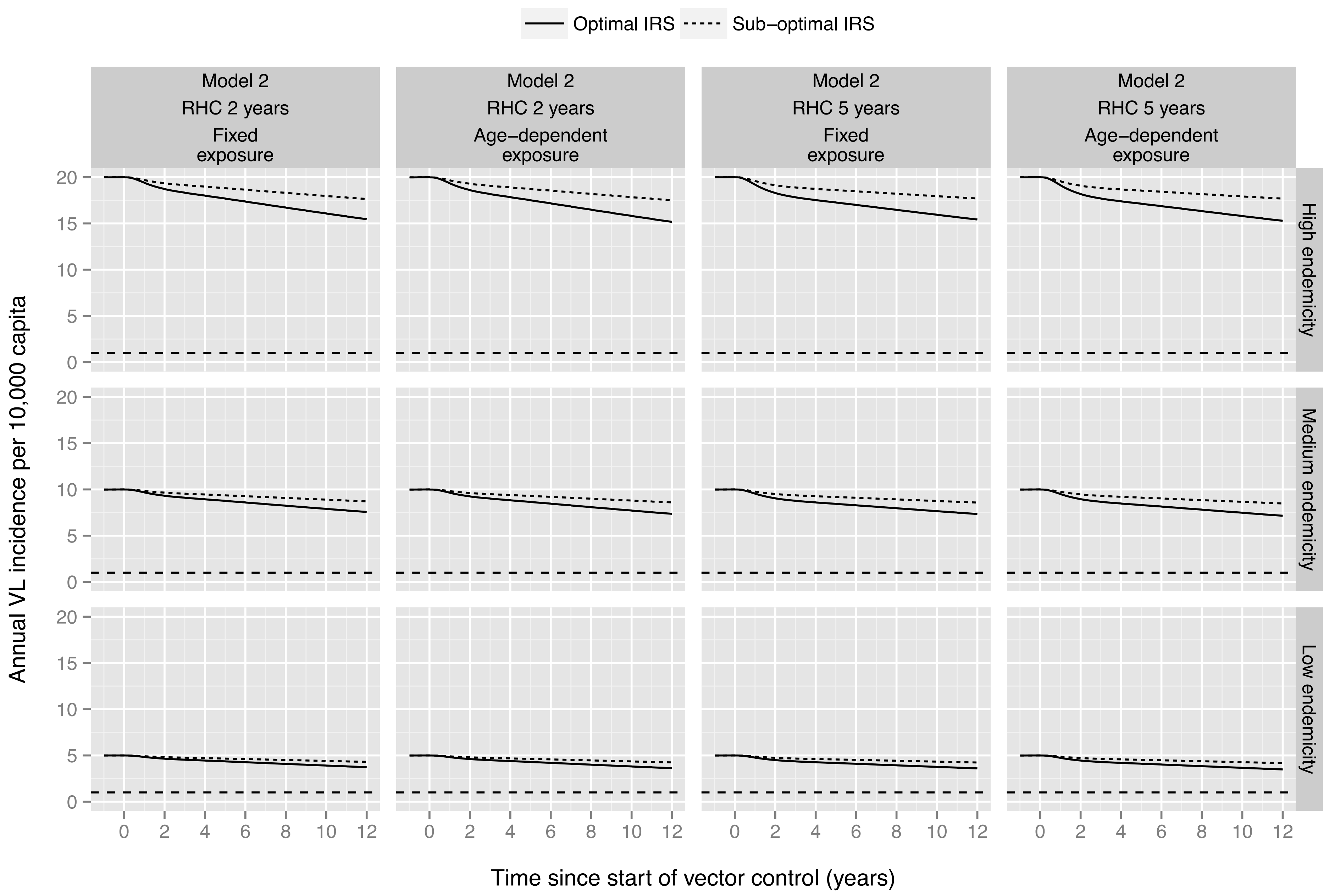

Optimal IRS
Sub−optimal IRS
Model 2
RHC 2 years
Fixed exposure
Model 2
RHC 2 years
Age−dependent exposure
Model 2
RHC 5 years
Fixed exposure
Model 2
RHC 5 years
Age−dependent exposure
High endemicity
Medium endemicity
Low endemicity
Annual VL incidence per 10,000 capita
Time since start of vector control (years)
0
5
10
15
20
0
2
4
6
8
10
12

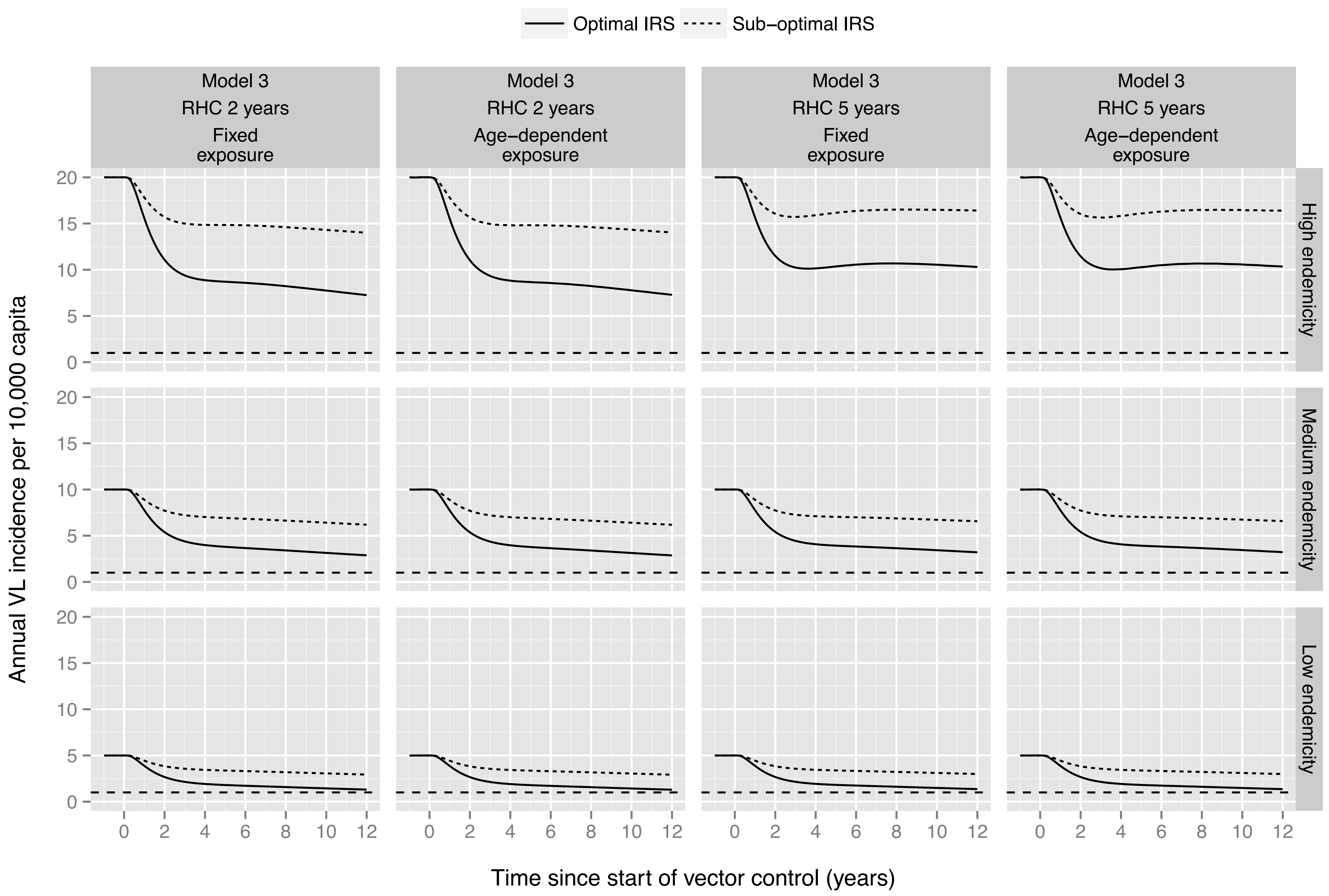
Optimal IRS
Sub−optimal IRS
Model 3
RHC 2 years
Fixed exposure
Model 3
RHC 2 years
Age−dependent exposure
Model 3
RHC 5 years
Fixed exposure
Model 3
RHC 5 years
Age−dependent exposure
High endemicity
Medium endemicity
Low endemicity
Annual VL incidence per 10,000 capita
Time since start of vector control (years)
0
5
10
15
20
0
2
4
6
8
10
12

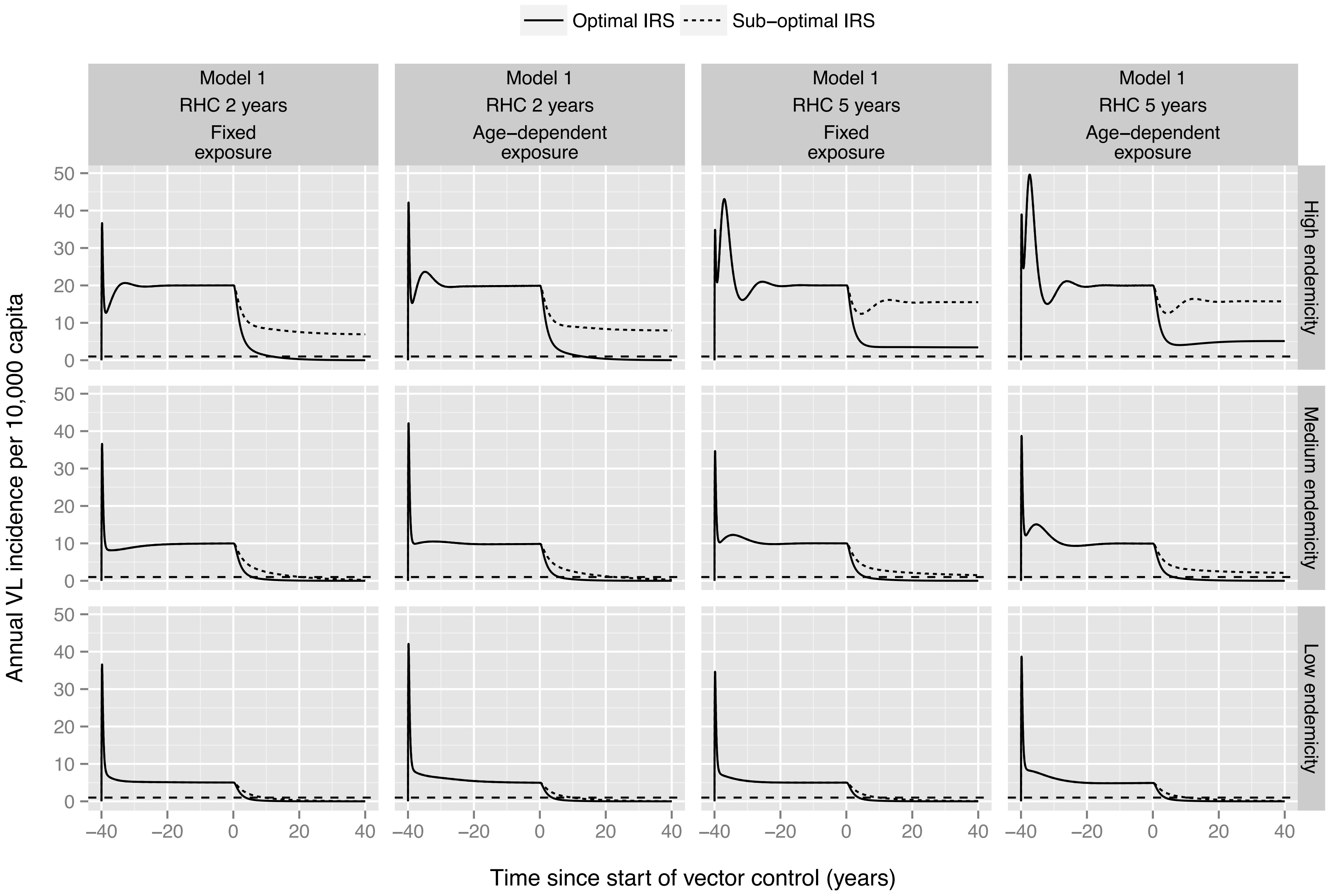
Optimal IRS
Sub−optimal IRS
Model 1
RHC 2 years
Fixed exposure
Model 1
RHC 2 years
Age−dependent exposure
Model 1
RHC 5 years
Fixed exposure
Model 1
RHC 5 years
Age−dependent exposure
High endemicity
Medium endemicity
Low endemicity
Annual VL incidence per 10,000 capita
Time since start of vector control (years)
0
10
20
30
40
50
−40
−20
0
20
40

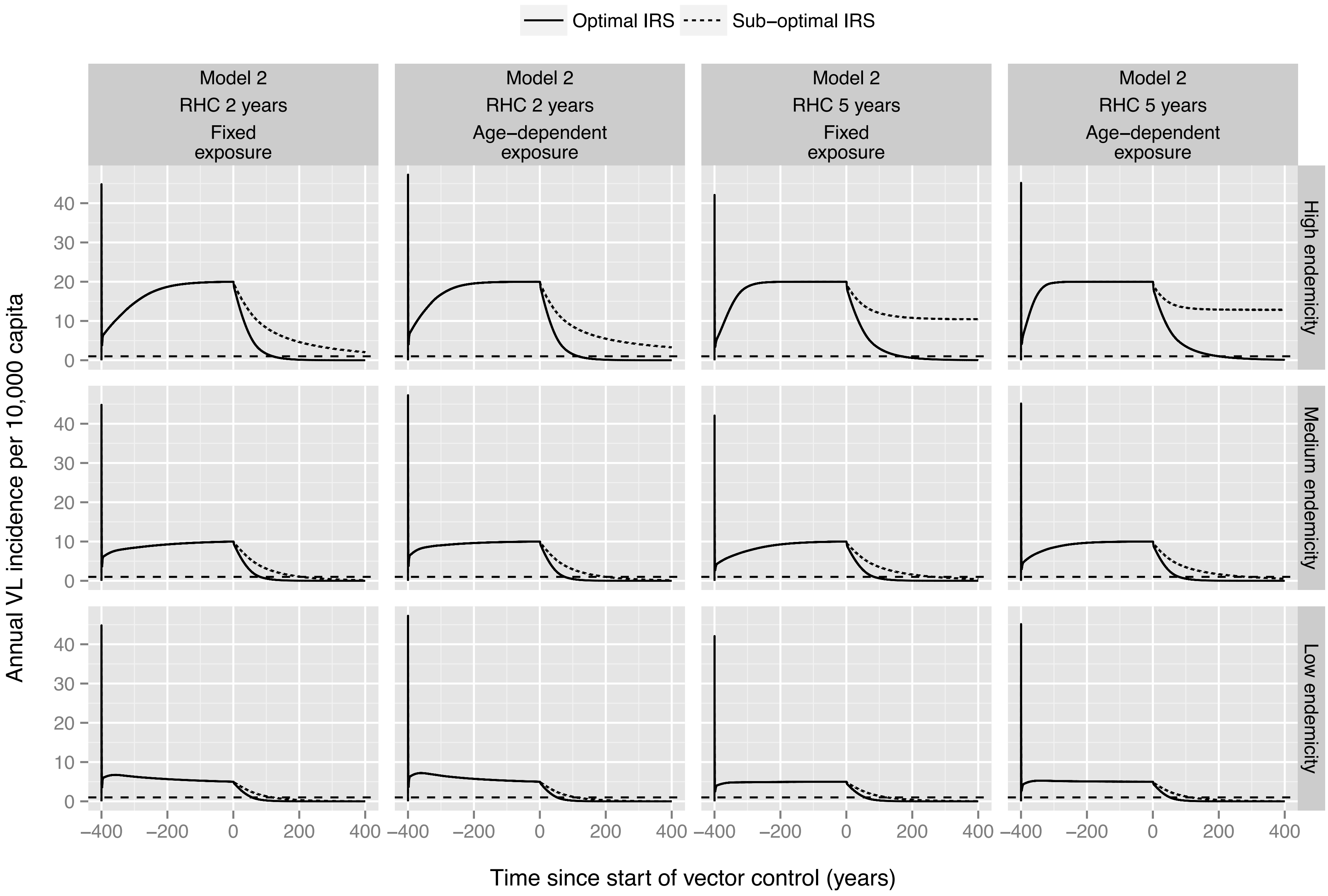

Optimal IRS
Sub−optimal IRS
Model 2
RHC 2 years
Fixed exposure
Model 2
RHC 2 years
Age−dependent exposure
Model 2
RHC 5 years
Fixed exposure
Model 2
RHC 5 years
Age−dependent exposure
High endemicity
Medium endemicity
Low endemicity
Annual VL incidence per 10,000 capita
Time since start of vector control (years)
0
10
20
30
40
−400
−200
0
200
400

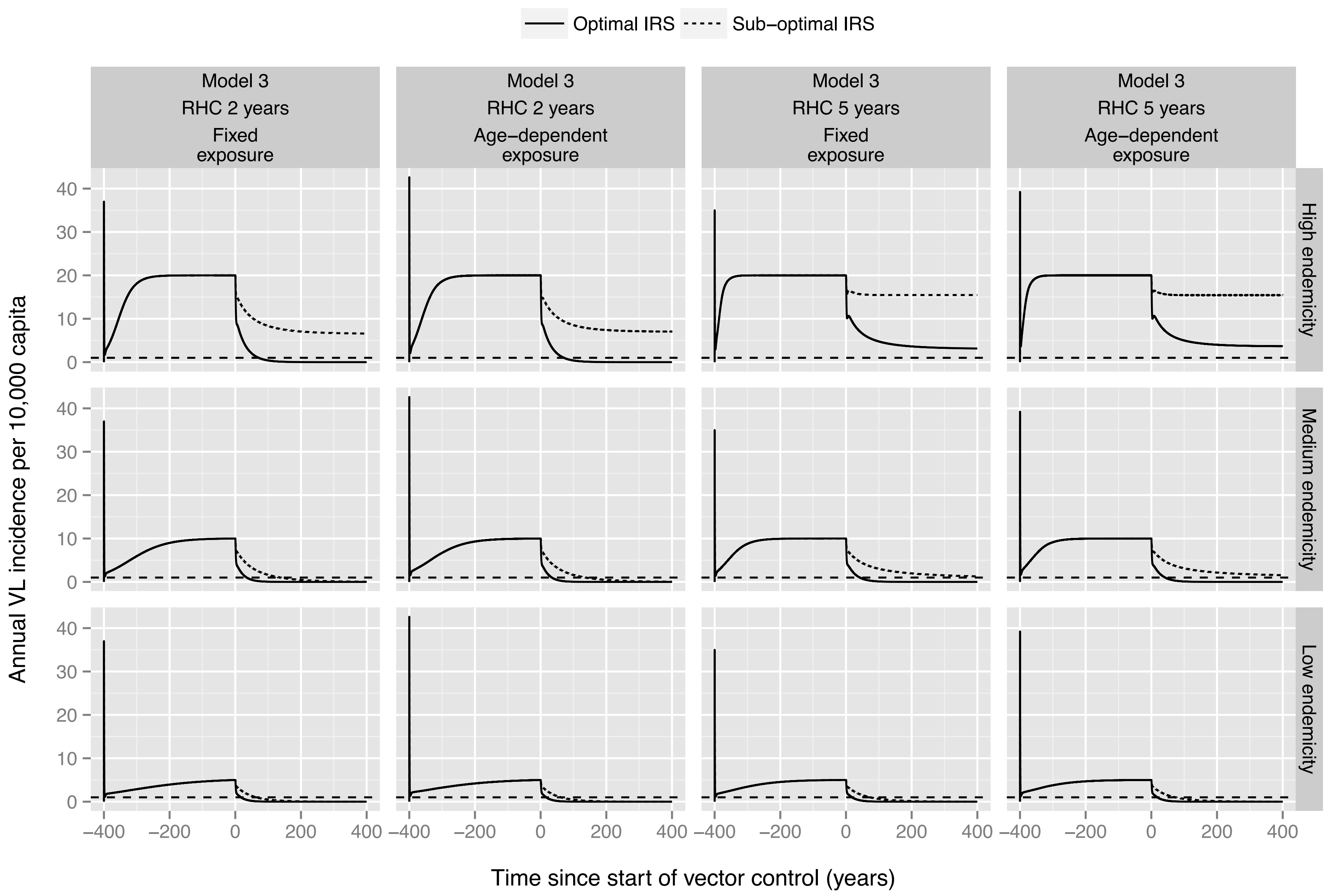

Optimal IRS
Sub−optimal IRS
Model 3
RHC 2 years
Fixed exposure
Model 3
RHC 2 years
Age−dependent exposure
Model 3
RHC 5 years
Fixed exposure
Model 3
RHC 5 years
Age−dependent exposure
High endemicity
Medium endemicity
Low endemicity
Annual VL incidence per 10,000 capita
Time since start of vector control (years)
0
10
20
30
40
−400
−200
0
200
400

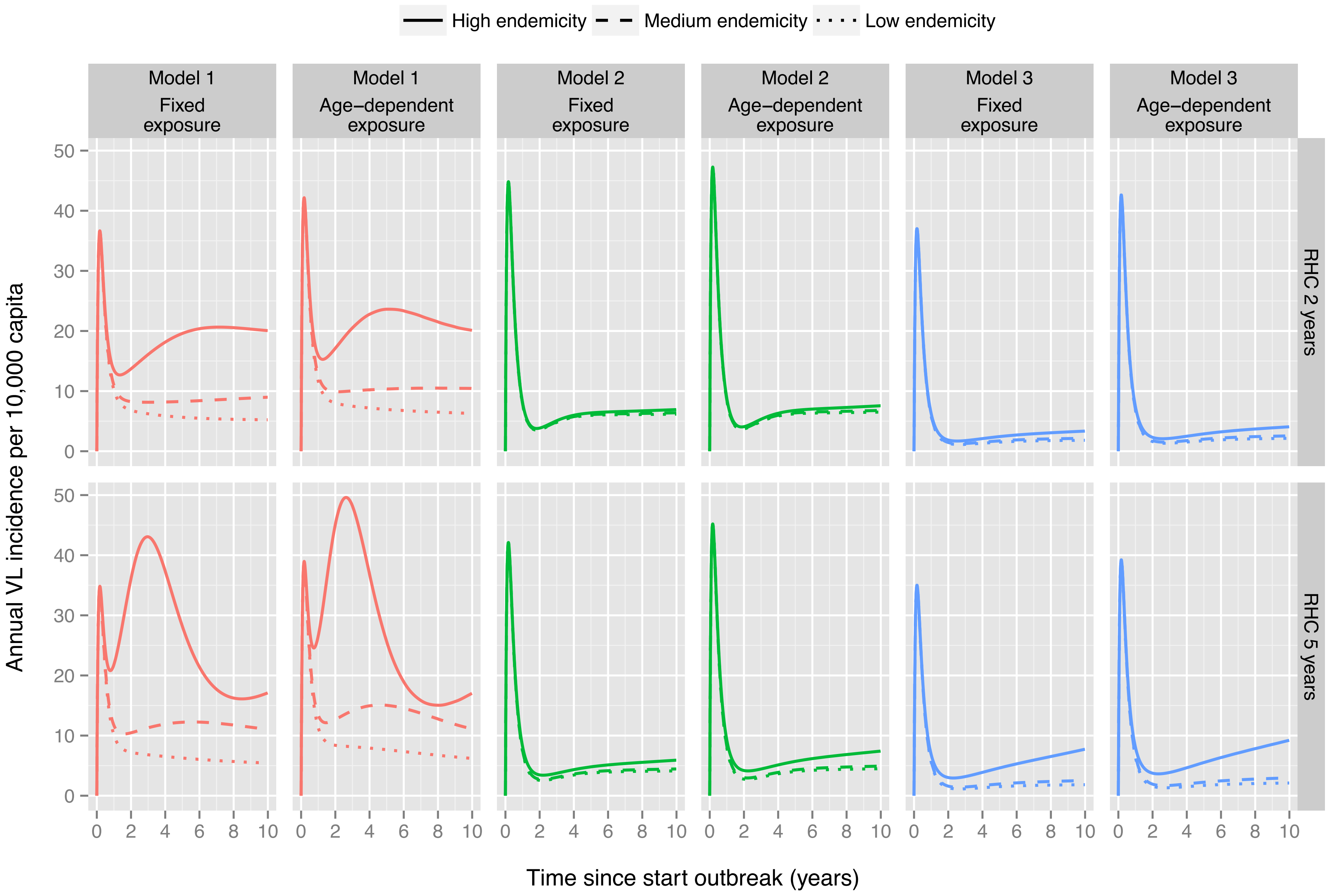

High endemicity
Medium endemicity
Low endemicity
Model 1 Fixed exposure
Model 1 Age−dependent exposure
Model 2 Fixed exposure
Model 2 Age−dependent exposure
Model 3 Fixed exposure
Model 3 Age−dependent exposure
RHC 2 years
RHC 5 years
Annual VL incidence per 10,000 capita
Time since start outbreak (years)
