# Supplementary figures and images for: Feasibility of eliminating visceral leishmaniasis from the Indian subcontinent: explorations with a set of deterministic age-structured transmission models
Source: Parasit Vectors. 2016 Jan 19;9:24. doi: 10.1186/s13071-016-1292-0 (PMC4717541; doi:10.1186/s13071-016-1292-0)

— High endemicity    - - Medium endemicity    ···· Low endemicity

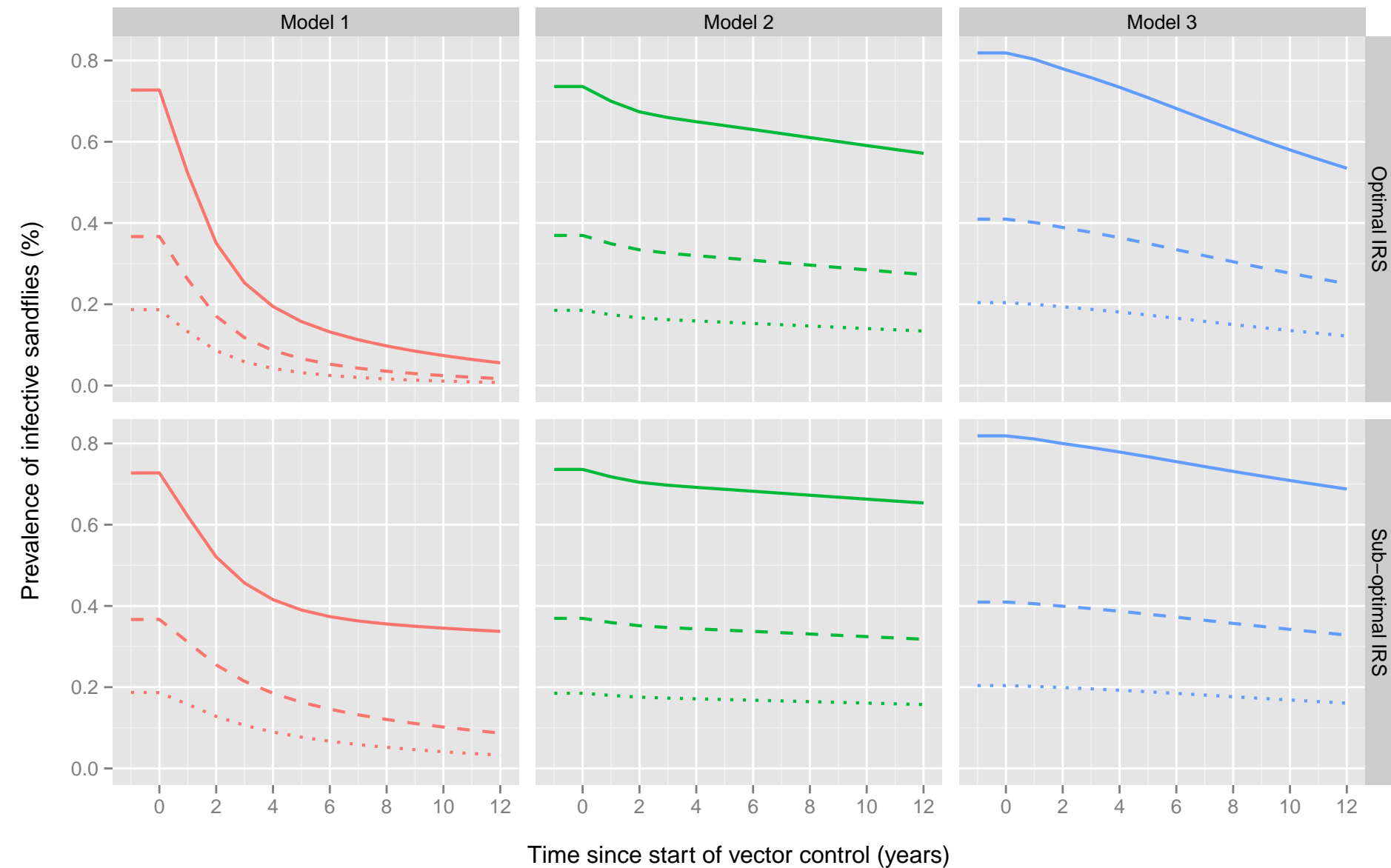

Supplement: Additional file 4: — Supplementary figure illustrating the impact of optimal IRS on the prevalence of infective sandflies in low, medium, and highly endemic settings, according to the best sub-variant of each model (extended version of Fig. 4 in the main manuscript). (PDF 7 kb) [file 13071_2016_1292_MOESM4_ESM.pdf]
