# Supplementary material for: Feasibility of eliminating visceral leishmaniasis from the Indian subcontinent: explorations with a set of deterministic age-structured transmission models
Source: Parasit Vectors. 2016 Jan 19;9:24. doi: 10.1186/s13071-016-1292-0 (PMC4717541; doi:10.1186/s13071-016-1292-0)
Supplement: Additional file 5: — Supplementary figure illustrating the impact of optimal IRS on incidence of VL in a sensitivity analysis of key estimated and assumed parameter values. (PDF 559 kb) [file 13071_2016_1292_MOESM5_ESM.pdf]

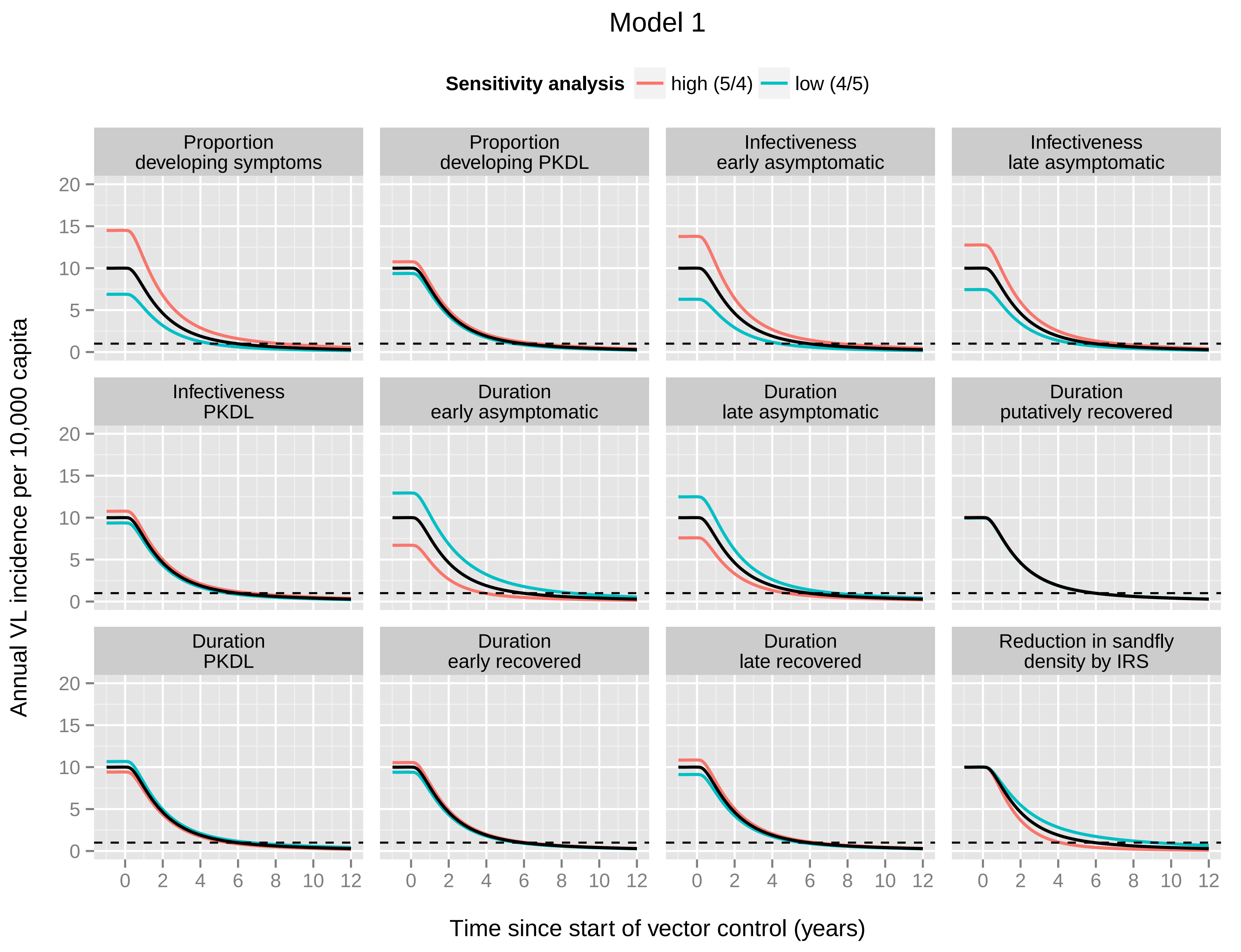

Model 1
Sensitivity analysis
high (5/4)
low (4/5)
Proportion developing symptoms
Proportion developing PKDL
Infectiveness early asymptomatic
Infectiveness late asymptomatic
Infectiveness PKDL
Duration early asymptomatic
Duration late asymptomatic
Duration putatively recovered
Duration PKDL
Duration early recovered
Duration late recovered
Reduction in sandfly density by IRS
Annual VL incidence per 10,000 capita
Time since start of vector control (years)
0
5
10
15
20
0
2
4
6
8
10
12

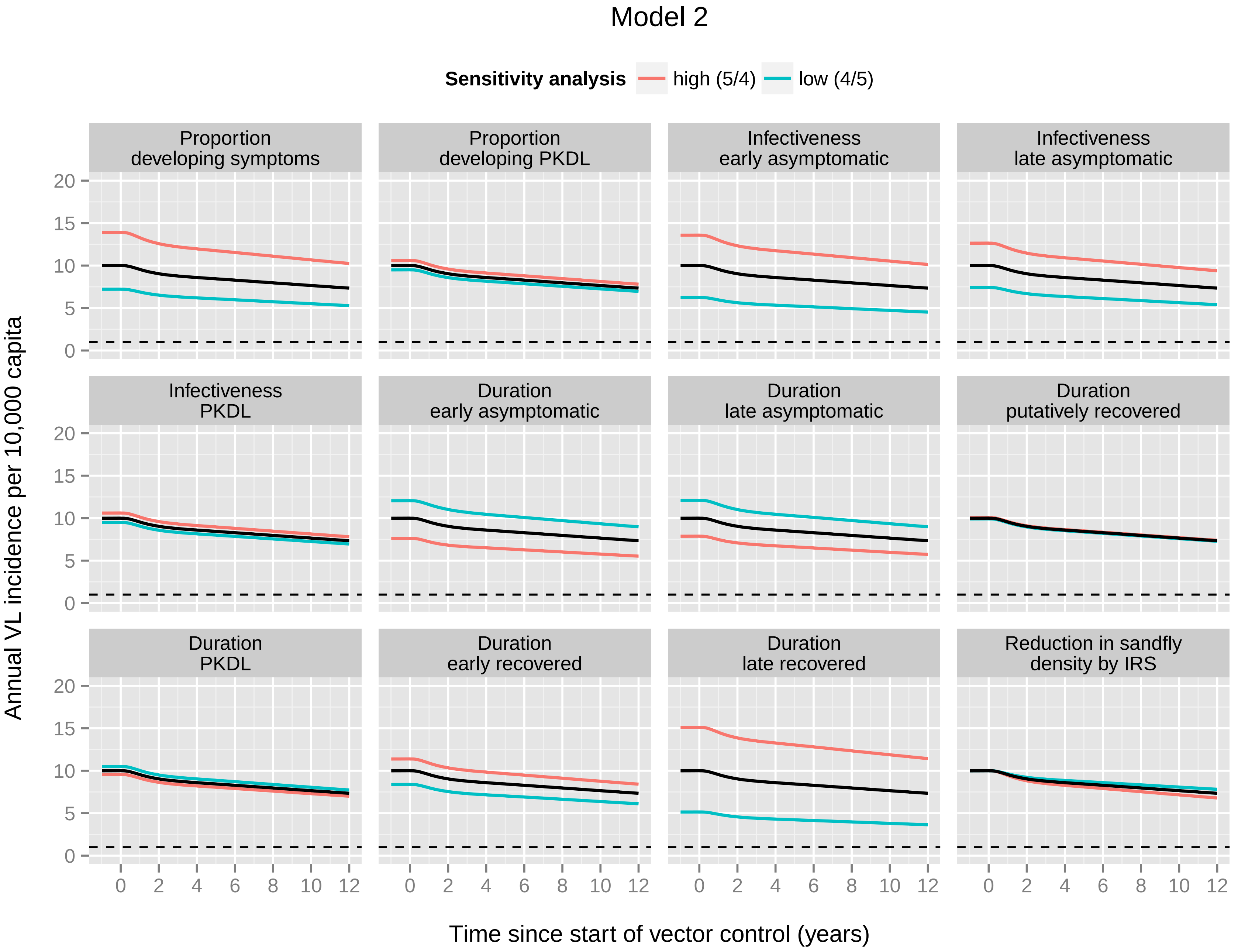
Model 2
Sensitivity analysis
high (5/4)
low (4/5)
Proportion developing symptoms
Proportion developing PKDL
Infectiveness early asymptomatic
Infectiveness late asymptomatic
Infectiveness PKDL
Duration early asymptomatic
Duration late asymptomatic
Duration putatively recovered
Duration PKDL
Duration early recovered
Duration late recovered
Reduction in sandfly density by IRS
Annual VL incidence per 10,000 capita
Time since start of vector control (years)
0
5
10
15
20
2
4
6
8
12

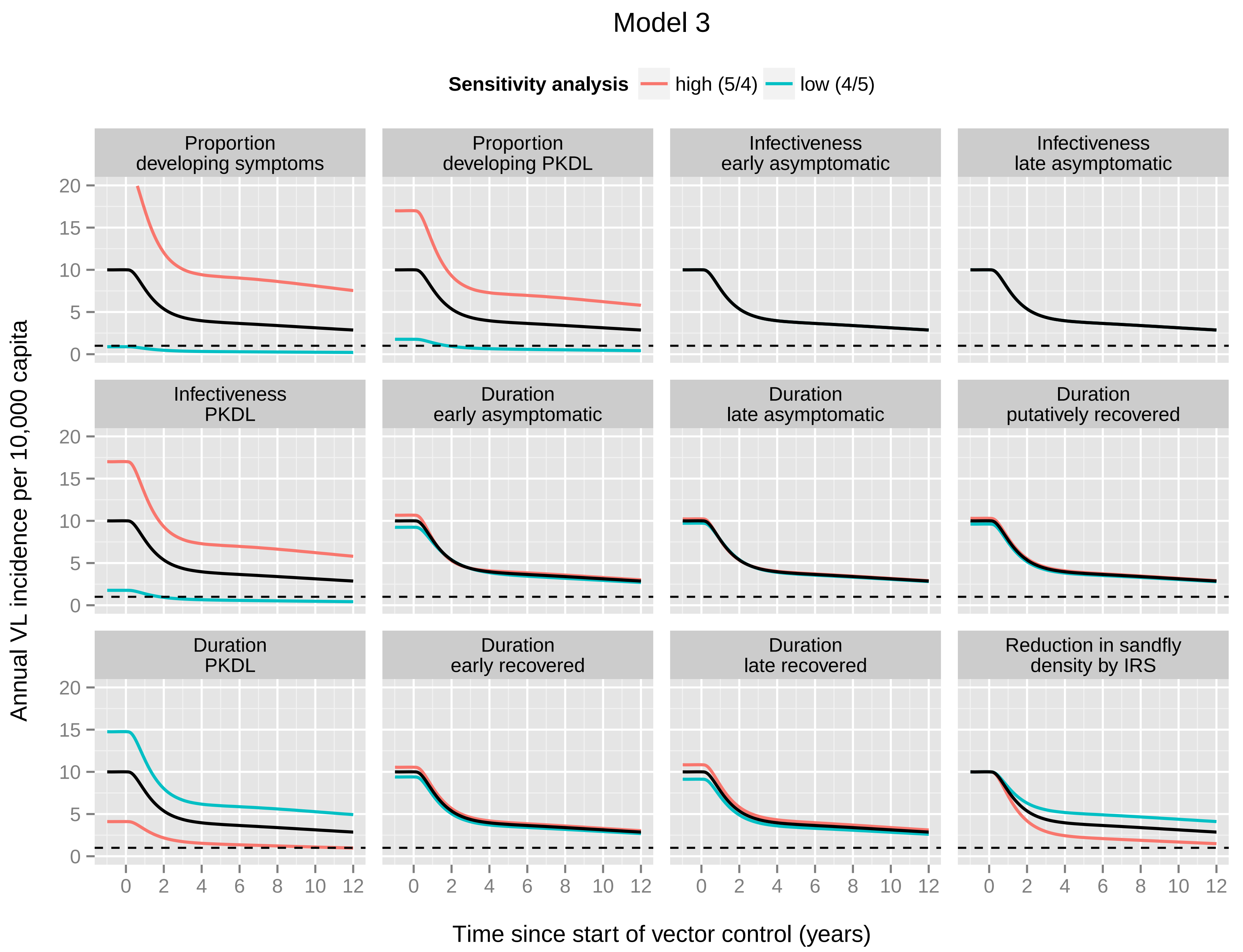

Model 3
Sensitivity analysis
high (5/4)
low (4/5)
Proportion developing symptoms
Proportion developing PKDL
Infectiveness early asymptomatic
Infectiveness late asymptomatic
Infectiveness PKDL
Duration early asymptomatic
Duration late asymptomatic
Duration putatively recovered
Duration PKDL
Duration early recovered
Duration late recovered
Reduction in sandfly density by IRS
Annual VL incidence per 10,000 capita
Time since start of vector control (years)
0
5
10
15
20
2
4
6
8
12

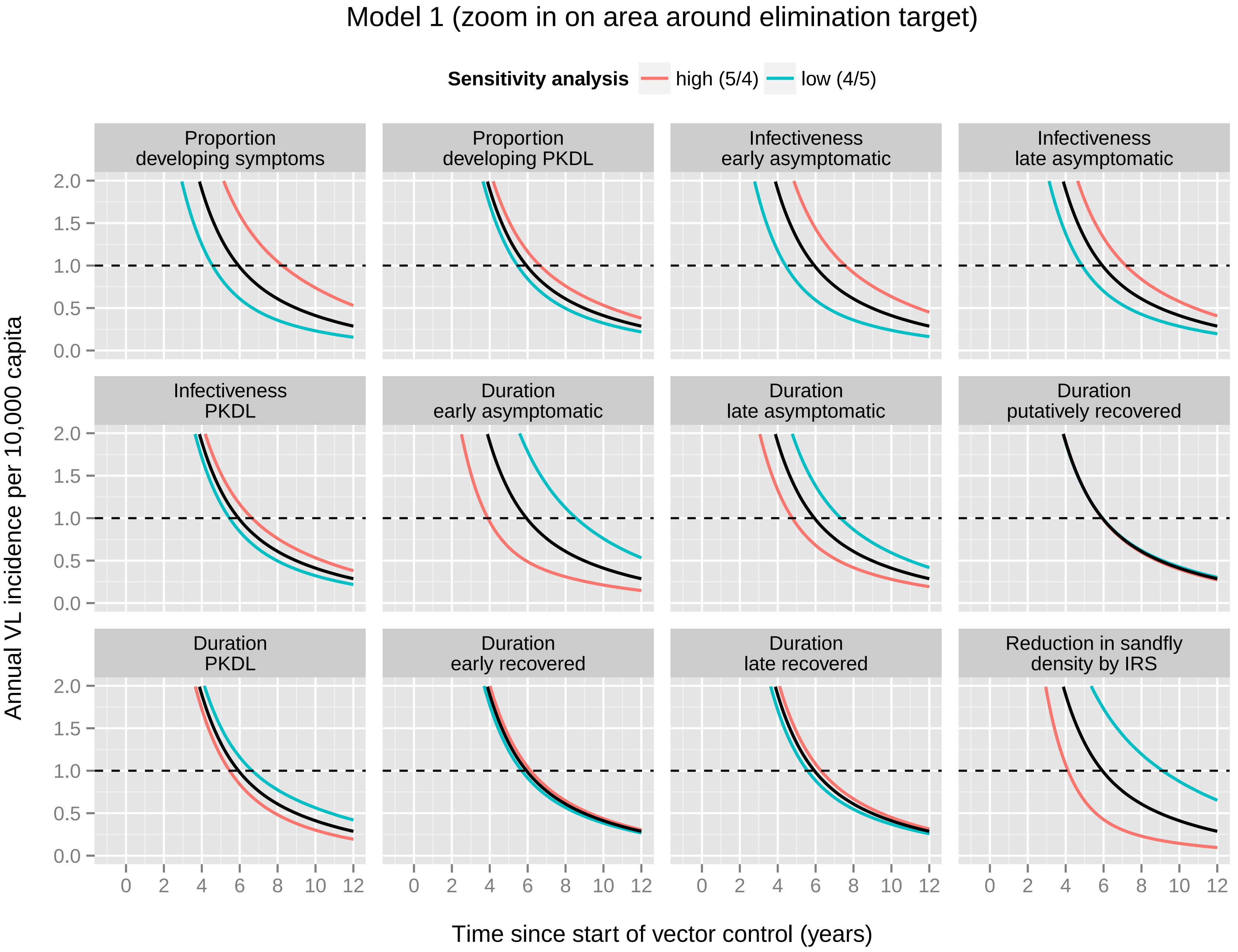

Model 1 (zoom in on area around elimination target)
Sensitivity analysis
high (5/4)
low (4/5)
Proportion developing symptoms
Proportion developing PKDL
Infectiveness early asymptomatic
Infectiveness late asymptomatic
Infectiveness PKDL
Duration early asymptomatic
Duration late asymptomatic
Duration putatively recovered
Duration PKDL
Duration early recovered
Duration late recovered
Reduction in sandfly density by IRS
Annual VL incidence per 10,000 capita
2.0
1.5
1.0
0.5
0.0
0
2
4
6
8
10
12
Time since start of vector control (years)
